# The protein deacetylase SIRT2 exerts metabolic control over adaptive **β** cell proliferation

**DOI:** 10.1172/JCI187020

**Published:** 2025-07-31

**Authors:** Matthew Wortham, Bastian Ramms, Chun Zeng, Jacqueline R. Benthuysen, Somesh Sai, Dennis P. Pollow, Fenfen Liu, Michael Schlichting, Austin R. Harrington, Bradley Liu, Thazha P. Prakash, Elaine C. Pirie, Han Zhu, Siyouneh Baghdasarian, Sean T. Lee, Victor A. Ruthig, Kristen L. Wells, Johan Auwerx, Orian S. Shirihai, Maike Sander

**Affiliations:** 1 Departments of Pediatrics and Cellular and Molecular Medicine, Pediatric Diabetes Research Center, UCSD, La Jolla, California, USA.; 2 Department of Pediatrics, Barbara Davis Center for Diabetes, University of Colorado Anschutz Medical Campus, Aurora, Colorado, USA.; 3 Institute of Chemistry and Biochemistry, Department of Biology, Chemistry and Pharmacy, Freie Universität Berlin, Berlin, Germany.; 4 Max Delbrück Center for Molecular Medicine in the Helmholtz Association, Berlin, Germany.; 5 Department of Antisense Drug Discovery, Ionis Pharmaceuticals Inc., Carlsbad, California, USA.; 6 Departments of Medicine and Molecular and Medical Pharmacology, David Geffen School of Medicine, UCLA, Los Angeles, California, USA.; 7 Developmental Biology Microscopy Consortium, Section of Developmental Biology of the Department of Pediatrics, and Department of Cell and Developmental Biology, University of Colorado Anschutz Medical Campus, Aurora, Colorado, USA.; 8 Laboratory of Integrated Systems Physiology, École Polytechnique Fédérale de Lausanne, Lausanne, Switzerland.

**Keywords:** Endocrinology, Metabolism, Beta cells, Diabetes, Mitochondria

## Abstract

Selective and controlled expansion of endogenous β cells has been pursued as a potential therapy for diabetes. Ideally, such therapies would preserve feedback control of β cell proliferation to avoid excessive β cell expansion. Here, we identified a regulator of β cell proliferation whose inactivation resulted in controlled β cell expansion: the protein deacetylase sirtuin 2 (SIRT2). *Sirt2* deletion in β cells of mice increased β cell proliferation during hyperglycemia with little effect under homeostatic conditions, indicating preservation of feedback control of β cell mass. SIRT2 restrains proliferation of human islet β cells, demonstrating conserved SIRT2 function. Analysis of acetylated proteins in islets treated with a SIRT2 inhibitor revealed that SIRT2 deacetylates enzymes involved in oxidative phosphorylation, dampening the adaptive increase in oxygen consumption during hyperglycemia. At the transcriptomic level, *Sirt2* inactivation has context-dependent effects on β cells, with *Sirt2* controlling how β cells interpret hyperglycemia as a stress. Finally, we provide proof of principle that systemic administration of a glucagon-like peptide 1–coupled (GLP1-coupled), *Sirt2*-targeting antisense oligonucleotide achieves β cell *Sirt2* inactivation and stimulates β cell proliferation during hyperglycemia. Overall, these studies identify a therapeutic strategy for increasing β cell mass in diabetes without circumventing feedback control of β cell proliferation. Future work should test the extent to which these findings translate to human β cells from individuals with or without diabetes.

## Abstract 2

The deacetylase SIRT2 restrains proliferation of pancreatic β-cells in a glucose-dependent manner, highlighting this enzyme as a potential therapeutic target for regenerating β-cells in diabetes

## Introduction

An insufficient mass of functional β cells underlies both type 1 and type 2 diabetes. Type 1 diabetes (T1D) results from autoimmune destruction of the majority of β cells, with residual β cells retaining some degree of function (1). Type 2 diabetes (T2D) predominantly involves dysfunction of β cells that have been challenged to sustain a high level of compensatory insulin secretion due to insulin resistance (2, 3). As the reduction of β cell mass is the principal cause of hyperglycemia in T1D, and β cell dysfunction in T2D is largely reversible by normalization of β cell workload (4), therapeutically increasing β cell mass through proliferation has been proposed for the treatment of both major types of diabetes.

Studies investigating therapeutic targets for expanding β cell mass have identified several compounds that stimulate proliferation of adult human β cells (5–8). Recent findings from several groups converged upon mechanisms involving inhibition of the kinase DYRK1A, with further increases of β cell proliferation achieved using combination treatment with DYRK1A inhibitors and other agents including glucagon-like peptide 1 (GLP1) (6) or inhibitors of the TGF-β pathway (5). The effectiveness of β cell expansion therapies in preclinical models generally supports the feasibility of this approach (6, 9), which is currently awaiting safety and efficacy results from clinical trials (10, 11).

Given the advances in achieving effective β cell expansion via experimental compounds, refinement of such approaches will address mitogenic effects in non–β cells (12) and the possibility of insulinoma formation or hypoglycemia. For example, a recent report of combined harmine and exendin-4 treatment of mice receiving human islet xenografts involved careful monitoring of β cell proliferation and the counterregulatory response to experimental hypoglycemia (13). Feedback control of cell expansion is a desirable feature of any cellular regeneration therapy. One mechanism controlling precise expansion of β cell mass in rodents is the glucose dependence of adaptive β cell proliferation (14), which couples β cell proliferation to increased β cell workload indicative of a shortfall of insulin. Preserving feedback regulation of proliferation during therapeutic β cell expansion would have the advantage of generating the precise number of β cells required for glycemic control.

Here, we identified a conserved mechanism that restrains β cell proliferation in a glucose-dependent manner: metabolic control of proliferation via the protein deacetylase sirtuin 2 (SIRT2). SIRT2 is a member of the sirtuin family of deacetylases that use nicotinamide adenine dinucleotide (NAD^+^) as a cosubstrate (15). Through genetic, pharmacological, and proteomics approaches, we show that SIRT2 is a suppressor of β cell proliferation at elevated glucose concentrations. In mice, SIRT2 inactivation enhanced β cell proliferation in vivo and ex vivo only at glucose concentrations above the normal glycemic set point, thereby preserving feedback control of β cell expansion. Preclinical studies using β cell–targeted *Sirt2* knockdown in vivo recapitulated this effect, establishing proof of principle for selectively and safely increasing β cell numbers.

## Results

### SIRT2 restrains the proliferation of mouse and human β cells ex vivo.

**Figure 1:**
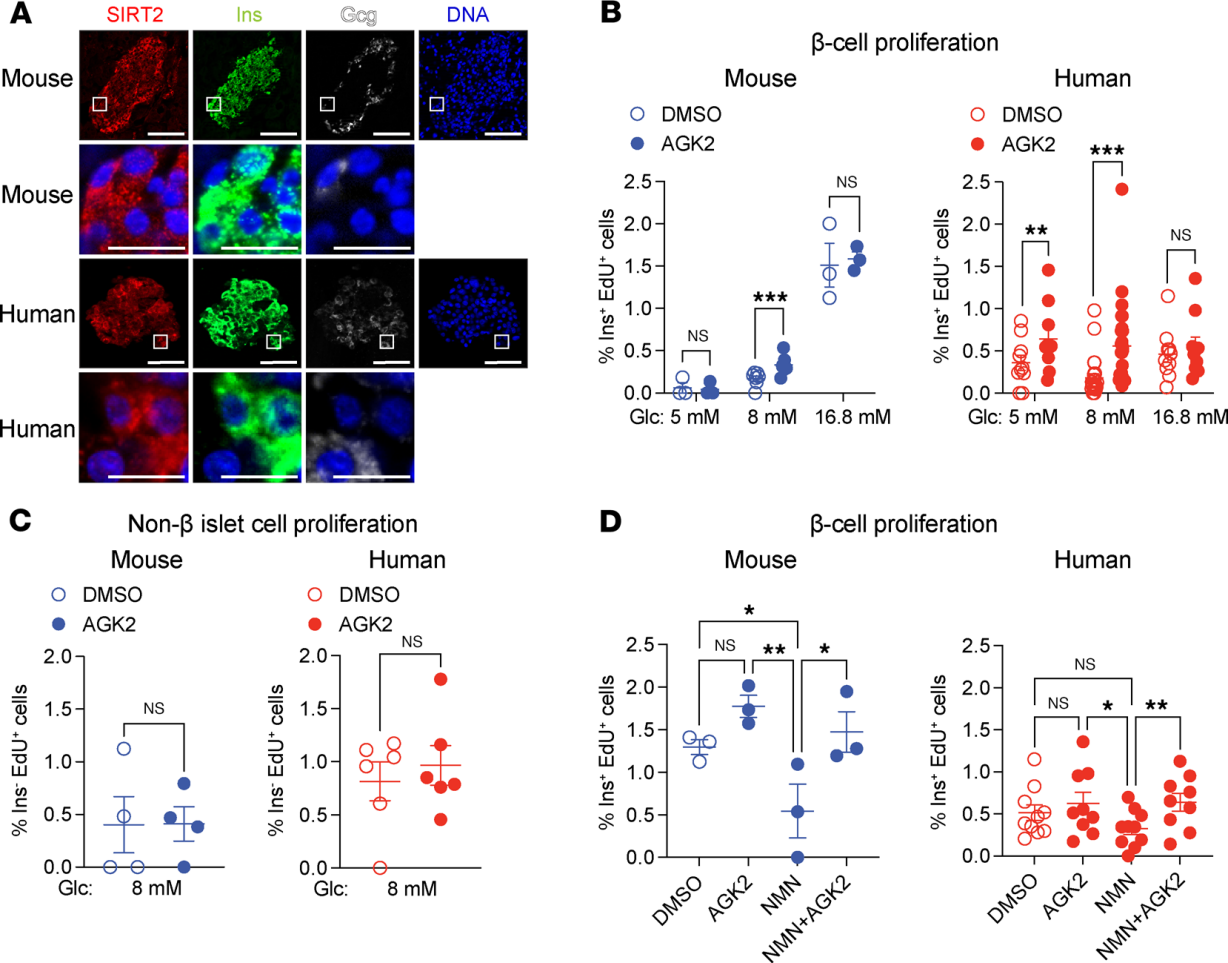
SIRT2 inhibition stimulates β cell proliferation in a glucose-dependent manner in cultured mouse and human islets. (**A**) Immunofluorescence staining for the indicated proteins in mouse pancreata and human islet sections. Enlarged images for the indicated areas, including DNA overlay, are shown below each channel. Scale bars: 50 µm (top) and 10 µm (enlarged images at bottom). Sections were counterstained for DNA using DAPI. Images are representative of 4 mouse pancreata and 2 human islet donors. (**B**) Quantification of β cell proliferation as a percentage of insulin-expressing cells positive for EdU in isolated mouse (blue, *n* = 3–7 islet preparations/group) and human (red, *n* = 10–25 islet preparations/group) islets after DMSO or AGK2 treatment during culture with the indicated glucose concentrations. (**C**) Quantification of non–β islet cell proliferation as a percentage of insulin^–^EdU^+^ cells in isolated mouse (blue, *n* = 4 islet preparations/group) and human (red, *n* = 6 islet preparations/group) islets after DMSO or AGK2 treatment. (**D**) Quantification of β cell proliferation as a percentage of insulin-expressing cells positive for EdU in isolated mouse (blue, *n* = 3 islet preparations/group) and human (red, *n* = 9–10 islet preparations/group) islets after treatment with DMSO, AGK2, NMN (an NAD^+^ precursor), or combinations thereof at 16.8 mM glucose. Data are shown as the mean ± SEM. Statistical differences were calculated using a multiple paired, 2-tailed Student’s *t* test (**B**) or a paired, 2-tailed Student’s *t* test (**C**) to determine statistical differences between 2 groups. A 1-way ANOVA with Tukey post hoc analysis was performed to analyze statistical differences between 3 or more groups (**D**). *P < 0.05, **P < 0.01, and ***P < 0.001. Glc, glucose; Ins, insulin.

β Cell proliferation in the postnatal period underlies developmental expansion of β cells in both rodents and humans. This process generates sufficient β cell mass for controlling glycemia in adulthood, at which point β cell proliferation declines precipitously (16, 17). Following developmental maturation, rodent but not human β cells maintain the capacity for adaptive proliferation in response to perceived insulin deficits. While this adaptive response is robust in young (1- to 2-month-old) animals, adaptive β cell proliferation is progressively attenuated with age (18–20). Given the age dependence of both basal and adaptive β cell proliferation, we reasoned that mechanisms controlling proliferation could be identified through assessment of age-regulated features of the β cell. To this end, we performed quantitative in vivo proteomics of islets from 1-month-old and 1-year-old mice (21), corresponding to ages with starkly different capacities for adaptive β cell proliferation (18, 20) and rates of basal β cell proliferation (22). Of differentially abundant proteins, we selected those with established roles in proliferation for follow-up studies, initially focusing on SIRT2 on the basis of its known role in suppressing the proliferation of immune cells and of various transformed cell types (23, 24). SIRT2 protein was elevated in islets from 1-year-old compared with 1-month-old mice (Supplemental Figure 1A; supplemental material available online with this article; https://doi.org/10.1172/JCI187020DS1), indicating an inverse association between SIRT2 abundance and capacity for β cell proliferation. Immunofluorescence staining identified SIRT2 in β cells in mouse pancreas and human islets (Figure 1A). The presence of SIRT2 in mouse and human β cells as well as its age-dependent increase in abundance led us to predict that SIRT2 is a conserved regulator of β cell proliferation.

To test whether SIRT2 regulates β cell proliferation, we used a pharmacological approach in isolated islets. As rodent β cell proliferation is coupled to glucose concentration (25), cultured mouse islets provide a means of assessing potential roles of SIRT2 in basal and adaptive β cell proliferation, with elevated glucose mimicking increased β cell workload. Mouse islets were incubated in the presence of increasing glucose concentrations together with the SIRT2-specific inhibitor AGK2 (26), and β cell proliferation was quantified (Figure 1B). AGK2 treatment increased β cell proliferation during culturing in 8 mM glucose, but not in 5 mM or 16.8 mM glucose (Figure 1B), indicating that SIRT2 inhibited β cell proliferation in a glucose concentration–dependent manner. To determine whether this function of SIRT2 is conserved, we measured β cell proliferation of AGK2-treated human islets, revealing a proproliferative effect of SIRT2 inhibition at both basal (5 mM) and intermediate (8 mM) glucose (Figure 1B) levels, despite the fact that glucose itself has little effect on human β cell proliferation, as expected (27–29). As SIRT2 protein is not uniquely expressed in β cells, we determined the cell type specificity of SIRT2’s effect on islet cell proliferation by quantifying the proliferation of insulin^–^ islet cells and found little effect of SIRT2 inhibition on non–β cells (Figure 1C). Previous studies have suggested that artificially stimulating β cell proliferation could cause DNA damage or impair insulin secretion (30, 31). However, AGK2 did not alter glucose-stimulated insulin secretion or insulin content of mouse or human islets (Supplemental Figure 1, B and C), nor did it activate the DNA damage response, as indicated by immunostaining for γH2AX (histone H2A.X phosphorylated at serine 139) (Supplemental Figure 1D). The inhibitory effect of SIRT2 on β cell proliferation in human islets was reproduced by 2 additional SIRT2-specific inhibitors, AK-1 (26) and SirReal2 (32) (Supplemental Figure 1E). Altogether, these results demonstrate that pharmacological SIRT2 inhibition increased mouse and human β cell proliferation without interfering with β cell function or affecting the proliferation of other islet cell types.

SIRT2 inactivation exerts a glucose-dependent effect on β cell proliferation, suggesting potential coupling between cellular metabolism and SIRT2 function. The requirement of SIRT2 for oxidized NAD^+^ as a cosubstrate together with the inhibitory effect of NADH (33) upon SIRT2’s enzymatic activity provide plausible mechanistic links between glucose metabolism and SIRT2-mediated dampening of proliferation. High-glucose exposure accelerates glycolysis in β cells, which generates NADH at the expense of NAD^+^. We therefore predicted that islet culture in 16.8 mM glucose sufficiently depletes NAD^+^ to inactivate SIRT2, rendering pharmacological SIRT2 inhibition of little effect. To test this, we performed rescue experiments to replenish NAD^+^ in high glucose using supplementation with the NAD^+^ precursor nicotinamide mononucleotide (NMN), and then determined the effect of SIRT2 inhibition on β cell proliferation. In mouse islets, NMN supplementation alone decreased β cell proliferation compared with DMSO- or AGK2-treated islets without NMN supplementation, suggesting that NAD^+^ depletion was necessary for maximum β cell proliferation in high glucose (Figure 1D). Importantly, SIRT2 inhibition via AGK2 in the context of NMN supplementation increased β cell proliferation over islets treated with NMN alone, suggesting that 16.8 mM glucose inactivated SIRT2 via NAD^+^ depletion (Figure 1D). Taken together, these results show that SIRT2 modulated β cell proliferation in a NAD^+^-dependent manner in both human and mouse islets, thereby coupling cellular metabolism to the control of proliferation by SIRT2.

### Sirt2 deletion enhances β cell proliferation under hyperglycemic conditions in vivo.

**Figure 2:**
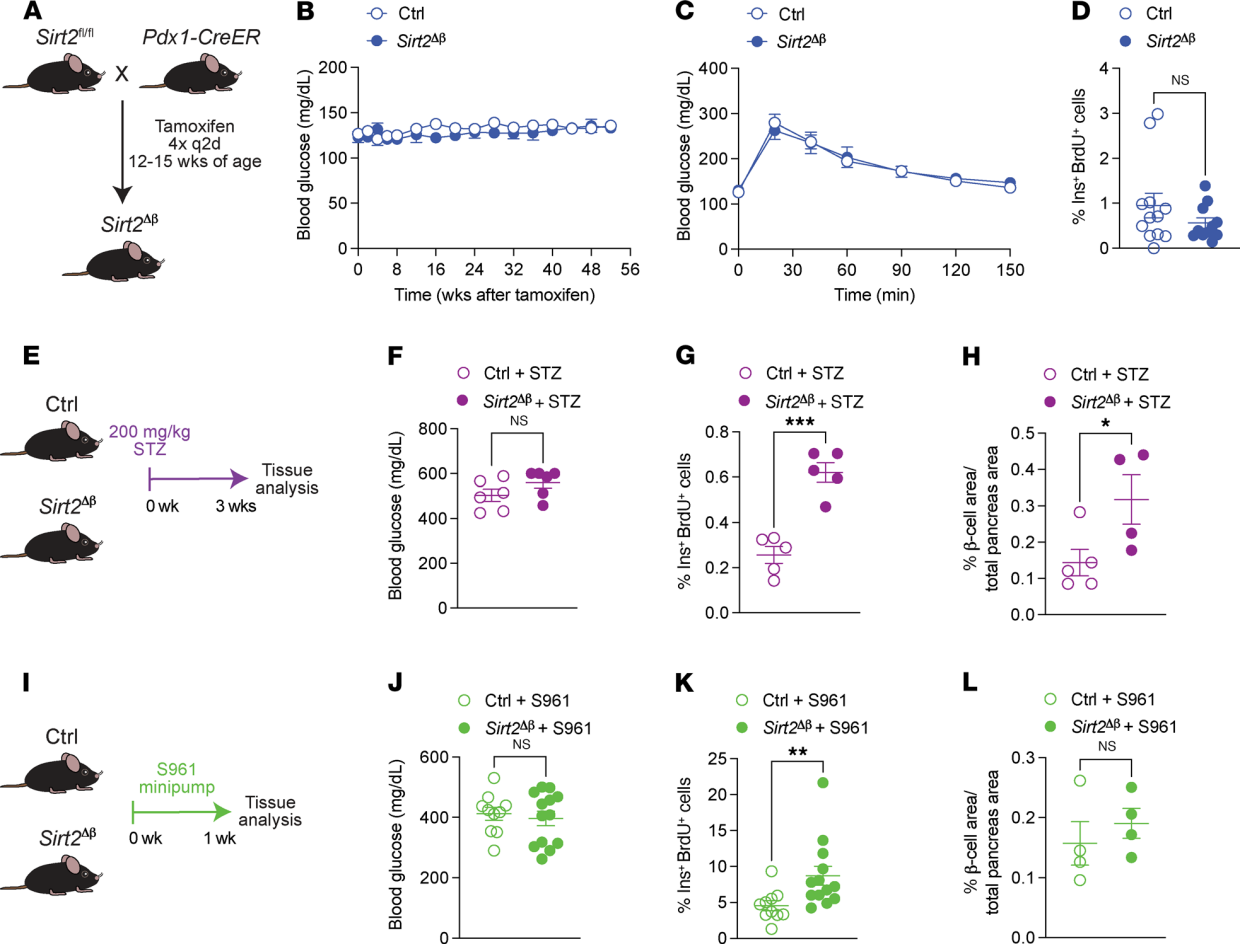
*Sirt2* inactivation enhances β cell proliferation in hyperglycemic conditions in vivo. (**A**) β Cell–selective *Sirt2*-KO mice (hereafter referred to as *Sirt2^Δβ^*) were generated by tamoxifen injection of 12- to 15-week-old *Sirt2^fl/fl^* *Pdx1CreER* mice. Tamoxifen-treated *Sirt2^+/+^* *Pdx1CreER* and *Sirt2^fl/+^* *Pdx1CreER* mice were used as controls. (**B**) Blood glucose levels measured for 52 weeks after tamoxifen treatment (*n* = 5–9 mice/group). (**C**) Blood glucose levels at the indicated time points following an intraperitoneal glucose injection, 52 weeks after tamoxifen treatment (*n* = 5–9 mice/group). (**D**) Quantification of β cell proliferation as a percentage of insulin^+^ and BrdU^+^ cells relative to total insulin^+^ cells, 4–6 weeks following tamoxifen treatment (*n* = 11–12 mice/group). (**E**) Hyperglycemia was induced in control and *Sirt2^Δβ^* mice by intraperitoneal injection of STZ (200 mg/kg body weight). After 3 weeks, (**F**) blood glucose levels (*n* = 6 mice/group), (**G**) β cell proliferation (*n* = 5 mice/group, and (**H**) relative β cell area (*n* = 4–5 mice/group) were measured. (**I**) Hyperglycemia was induced in control and *Sirt2^Δβ^* mice by administering S961 via transplanted minipumps (20 nmol/week). After 1 week, (**J**) blood glucose levels (*n* = 10–13 mice/group), (**K**) β cell proliferation (*n* = 10–13 mice/group), and (**L**) β cell area (*n* = 4 mice/group) were measured. Data are shown as the mean ± SEM. Statistical differences were calculated using a 2-way ANOVA with Tukey’s post hoc analysis (**B** and **C**) or an unpaired, 2-tailed Student’s *t* test (**D**, **F**–**H**, and **J**–**L**). *P < 0.05, **P < 0.01, and ***P < 0.001. q2d, every 2 days; Ctrl, control.

The role of SIRT2 in restraining β cell proliferation ex vivo, together with the age-dependent increase in SIRT2 abundance, led us to predict that SIRT2 could inhibit the proliferation of adult β cells. To test this, we conditionally inactivated *Sirt2* in β cells of adult *Sirt2^fl/fl^* mice using the tamoxifen-inducible *Pdx1CreER* transgene (*Sirt2*^Δβ^ mice hereafter) and then monitored glucose homeostasis (Figure 2A and Supplemental Figure 2A). Periodic glucose tolerance tests and measurements of ad libitum blood glucose levels over the course of 1 year revealed no evidence of altered glycemia or glucose tolerance in *Sirt2*^Δβ^ mice (Figure 2, B and C, and Supplemental Figure 2, B and C), consistent with ex vivo data indicating that SIRT2 inactivation was of little effect on mouse islets under normoglycemic conditions (Figure 1B and Supplemental Figure 1B). Moreover, markers of β cell identity, Pdx1 and Nkx6.1, were unchanged in β cells of *Sirt2*^Δβ^ mice compared with those of control mice (Supplemental Figure 2, E and F). Quantification of β cell proliferation and β cell area showed no differences between *Sirt2*^Δβ^ and control mice (Figure 2D and Supplemental Figure 2D). Similar results were obtained following *Sirt2* deletion using the *MIP-CreER* transgene (Supplemental Figure 2, G–K). Overall, these results indicate that SIRT2 does not regulate basal β cell proliferation or function under homeostatic conditions.

As we found that SIRT2 regulates β cell proliferation in a glucose-dependent manner ex vivo (Figure 1B), we hypothesized that the effect of *Sirt2* deletion on β cell proliferation in vivo would be contingent on hyperglycemia. To test this, we injected mice with a single high dose of streptozotocin (STZ) (Figure 2, E and F) and quantified β cell proliferation and relative β cell area. We observed increased β cell proliferation (Figure 2G) and pancreatic insulin^+^ area (Figure 2H) in STZ-treated *Sirt2*^Δβ^ mice compared with STZ-treated control mice. Similar findings were obtained following *Sirt2* deletion using the *MIP*-*CreER* transgene (Supplemental Figure 2, L–N). While the proproliferative effect of *Sirt2* inactivation is consistent with the expected increase of the relative β cell area, we cannot rule out the effects of SIRT2 on β cell survival in response to STZ. To complement the β cell ablation studies, we used a second hyperglycemic model and administered the insulin receptor antagonist S961 to *Sirt2*^Δβ^ and control mice using micro-osmotic pumps to increase β cell workload without ablating β cells (Figure 2I) (34–36). After 1 week, the mice showed highly elevated basal blood glucose with no difference in blood glucose levels between *Sirt2*^Δβ^ and control mice (Figure 2J). S961 treatment stimulated a very high rate of β cell proliferation that was enhanced by β cell *Sirt2* deficiency (Figure 2K). *Sirt2* deletion did not increase the relative β cell area compared with S961-treated control mice (Figure 2L), possibly due to the short duration of hyperglycemia. Altogether, these observations indicate that SIRT2 had context-dependent effects on β cell proliferation in vivo, restraining adaptive proliferation during increased β cell workload but having a minimal effect on β cell turnover in basal conditions.

### SIRT2 deacetylates enzymes involved in oxidative metabolism of glucose and fatty acids.

**Figure 3:**
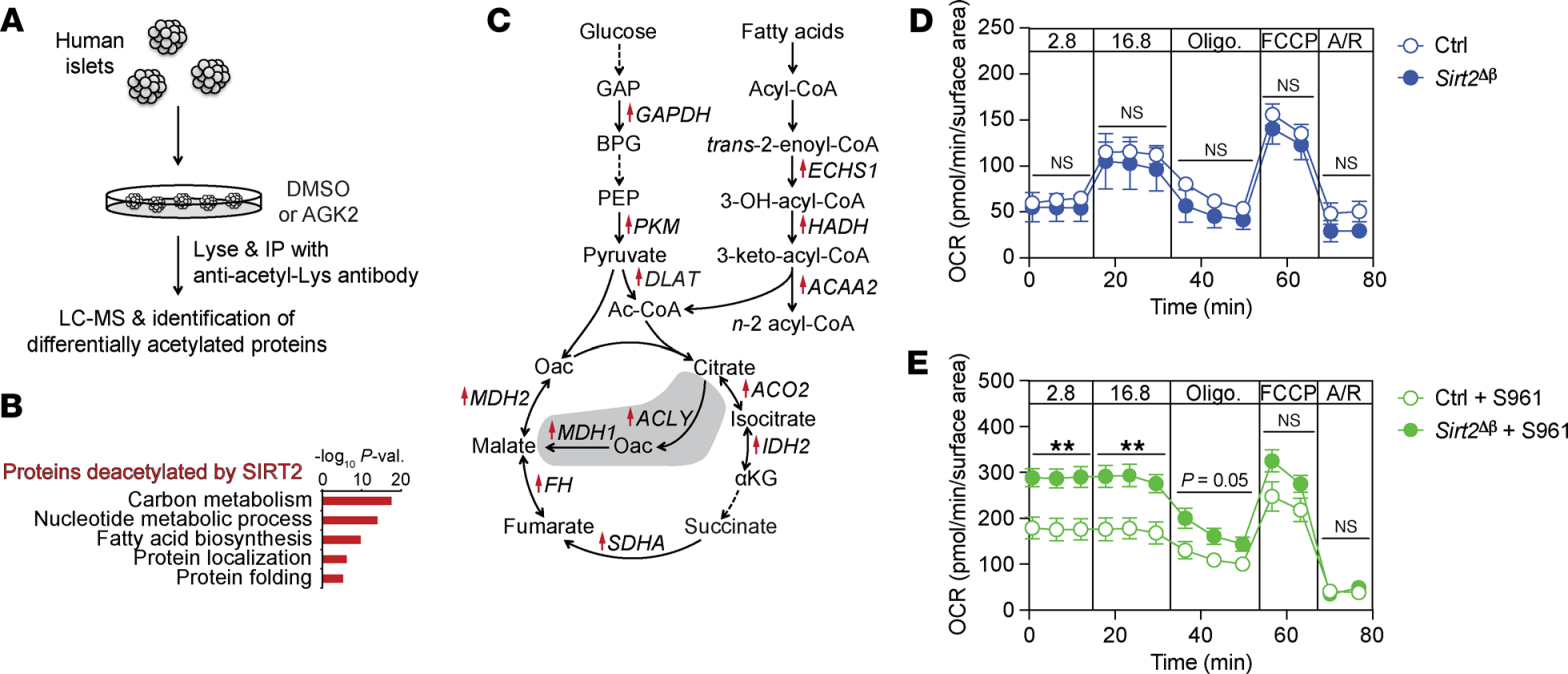
SIRT2 deacetylates metabolic enzymes and restrains islet OxPhos during hyperglycemia. (**A**) Schematic of proteomics experiment to identify proteins deacetylated by SIRT2 in human islets. (**B**) GO enrichment analysis of proteins exhibiting 1.5-fold or higher acetylation following AGK2 treatment compared with vehicle control, as detected by differential abundance following lysate enrichment using an antibody recognizing acetyl-lys. (**C**) Schematic of metabolic enzymes deacetylated by SIRT2. Proteins with increased acetyl-lys following AGK2 treatment are indicated with red arrows. Cytosolic reactions downstream of pyruvate are shaded. (**D** and **E**) OCR of islets from untreated (**D**) or S961-treated (**E**; as in Figure 2I) control and *Sirt2^Δβ^* mice treated sequentially with the indicated glucose concentrations (in mM), oligomycin (Oligo.), FCCP, and antimycin/rotenone (A/R). *n* = 5–6 pools of islets/group. The OCR was assessed using size-matched islets following 48 hours of culture in standard islet medium containing 8 mM glucose (**D**) or in freshly isolated islets (**E**). Tamoxifen-treated *Sirt2^+/+^ Pdx1CreER* and *Sirt2^fl/+^* *Pdx1CreER* mice were used as controls. Data are shown as the mean ± SEM. Statistical differences were calculated using a 2-way ANOVA for each time block. **P < 0.01 for genotype.

The above experiments demonstrate a key role for SIRT2 in dampening adaptive β cell proliferation. To identify potential mechanisms whereby SIRT2 regulates β cell proliferation, we sought to identify its deacetylation targets in β cells. While SIRT2’s targets have been assessed in some contexts (24, 37), it is unclear whether these substrates are cell-type or context dependent. We therefore cataloged proteins that are deacetylated by SIRT2 in human islets. To this end, we quantified SIRT2-dependent lysine acetylation proteome wide by treating human islets with the SIRT2 inhibitor AGK2, enriching islet lysates with an antibody recognizing acetylated lysine, and then performing mass spectrometry–based proteomics analysis of the enriched lysates (Figure 3A). Acetylated proteins exhibiting at least 1.5-fold greater abundance in the anti–acetyl-lysine fraction of AGK2-treated relative to DMSO-treated islet lysates (64 proteins) were considered deacetylation substrates of SIRT2 (Supplemental Table 1, A and B). SIRT2 targets did not include canonical cell-cycle regulators such as cyclins, cyclin-dependent kinase inhibitors, or signal transduction components downstream of known β cell mitogens (38). We therefore asked whether SIRT2 coordinately regulates cellular processes that could indirectly affect proliferation. Gene ontology (GO) analysis of SIRT2 substrates revealed enrichment of proteins involved in central carbon metabolism (Figure 3, B and C, and Supplemental Table 1C) including glycolytic enzymes (GAPDH and PKM), fatty acid β-oxidation enzymes (ACAA2 and HADH), and TCA enzymes (ACO2, FH, IDH2, MDH2, and SDHA). Several of these SIRT2 targets in islets agree with SIRT2 deacetylation targets in T lymphocytes (24), where it was shown that *Sirt2* deletion enhances the enzymatic activities of GAPDH, ACO2, and SDH to augment cell proliferation. The enhanced acetylation and consequent hyperactivation of these enzymes were suggested to contribute to increased oxidative metabolism following SIRT2 inactivation in T cells, leading us to predict that *Sirt2*^Δβ^ islets would be more oxidative than control islets. To test this possibility, we performed respirometry of *Sirt2*^Δβ^ and control islets isolated from both unchallenged and S961-treated animals. While β cell SIRT2 inactivation did not affect oxygen consumption in islets from untreated mice (Figure 3D), islets isolated from S961-treated *Sirt2*^Δβ^ animals consumed more oxygen than did islets from S961-treated control animals (Figure 3E). Together, these results indicate that SIRT2 deacetylates metabolic enzymes and regulates adaptive changes to islet metabolism.

### S961-induced insulin resistance and hyperglycemia remodel the β cell transcriptional state.

**Figure 4:**
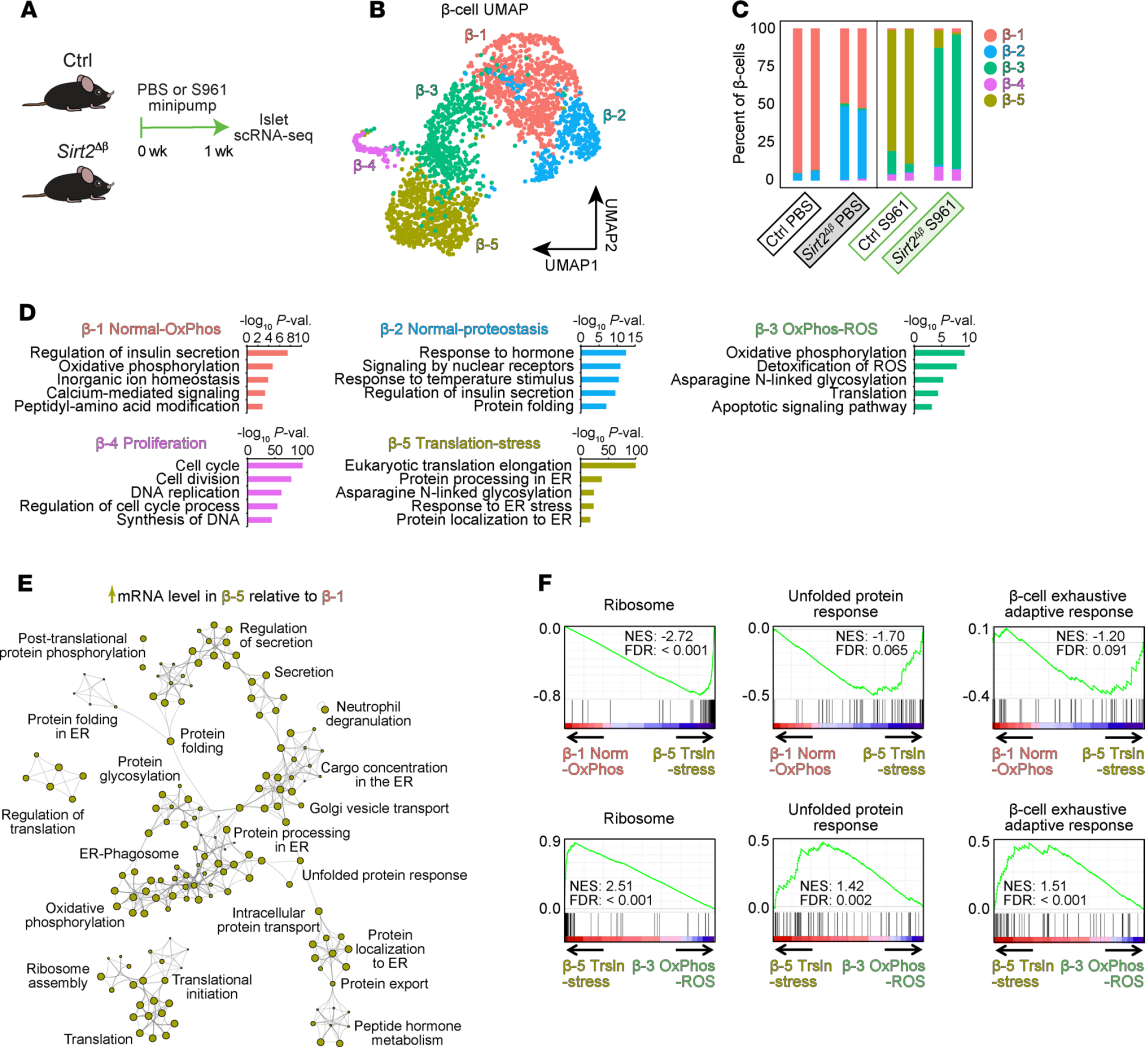
*Sirt2* inactivation affects the transcriptional response of β cells to hyperglycemia. (**A**) Schematic of the scRNA-Seq experiment. Tamoxifen-treated *Sirt2^+/+^* *Pdx1CreER* and *Sirt2^fl/+^* *Pdx1CreER* mice were used as controls. (**B**) Uniform manifold approximation and projection (UMAP) plot of clustered β cells colored by subset. (**C**) Abundance of each β cell subset as a proportion of total β cells for the indicated treatments and genotypes (*n* = 2 technical replicates of islets pooled from 2–3 mice per group). (**D**) Enriched gene ontologies and pathways for mRNAs more highly expressed in each subset relative to all other β cells (FDR < 0.05). val., value. (**E**) Network of GOs and pathways for mRNAs more highly expressed in β-5 cells compared with β-1 cells (FDR < 0.05). (**F**) GSEA of the indicated β cell stress response gene sets for genes ranked by FDR for pairwise comparisons between the indicated subsets of β cells. The FDR was determined by Wilcoxon rank-sum test for subset-specific genes (**D**) and pairwise comparisons of β cell subsets (**E** and **F**). NES, normalized enrichment score; norm, normal; trsln, translation.

The glucose dependence of SIRT2’s effect on β cell proliferation suggests that *Sirt2* deletion could affect how β cells interpret hyperglycemia. In addition to serving as a key signal for adaptive β cell proliferation, glucose can exert deleterious effects on β cells through maladaptive activation of stress response pathways. To study how β cells respond to hyperglycemic conditions in vivo, we performed single-cell RNA-Seq (scRNA-Seq) analysis of islets from mice treated with S961 for 1 week (Figure 4A and Supplemental Figure 3A). β Cells were identified on the basis of their expression of *Ins1* and *Ins2* and were reclustered for further analysis, revealing 5 β cell subsets with variable abundance across conditions (Figure 4, B and C). We first asked how S961 affects the composition of β cell subsets in control mice. In untreated control mice, β cells comprised 93%–95% β-1 cells and a minority population (4%–6%) of β-2 cells (Figure 4, B and C), which agrees with the extent of transcriptional heterogeneity previously described in homeostatic conditions (39, 40). The β cell subset composition was dramatically affected by S961 treatment, which shifted β cells to transcriptional subsets that were not detected in control islets. Islets from S961-treated mice comprised 80%–88% β-5 cells and minority populations of β-3 (5%–15%) and β-4 (4%–5%) cells. The rapidity of these changes to β cell subset composition far exceeded estimates of β cell turnover during 1 week of S961 treatment (41). Therefore, S961 caused β cells to shift to a distinct set of transcriptional states that were exceedingly rare in unchallenged conditions.

We reasoned that the above-described effects of S961 on β cell states likely reflected engagement of different cellular processes as β cells responded to the systemic effects of hyperglycemia. To predict the activation of cellular responses in each β cell subset, we analyzed genes highly expressed in each subset relative to all other β cells for enrichment of gene ontologies (GOs) and pathways from KEGG and Reactome databases (Figure 4D and Supplemental Table 2, A–F). As expected, the β cell subset comprising the majority population in control mice (β-1) exhibited a gene expression signature of insulin secretion as well as enrichment of GO terms including metabolic pathways and ion channels characteristic of normal β cells (Figure 4D). The minority population of β cells in unchallenged mice (β-2) was also characterized by a signature of insulin secretion with additional enrichment of genes involved in protein folding (Figure 4D), which we speculate relates to the previously described signature of transiently elevated protein processing in the endoplasmic reticulum (ER) (42). Considering the relative abundance of these subsets in unchallenged mice (Figure 4C), we designated β-1 and β-2 cells as normal–oxidative phosphorylation (normal-OxPhos) cells and normal-proteostasis cells, respectively. β-4 cells expressed genes involved in DNA replication and cell division, indicating that these β cells were proliferating, as has been well documented to occur in response to increased workload (14, 43). The remaining β cell subsets induced by S961 treatment (β-3 and β-5) overexpressed genes associated with both translation and protein glycosylation, with the majority-population β-5 cells further expressing a signature of protein processing in the ER and β-3 cells expressing a signature of OxPhos and ROS. β-3 and β-5 cells were therefore designated as OxPhos-ROS and translation-stress cells, respectively. Given the similarity of pathway enrichments for several β cell subsets, we directly compared transcriptomes between subsets to predict relative gene activities of pathways or cellular processes for each β cell state. To this end, we determined enrichment of GO terms and pathways among genes differentially expressed between states. To analyze the major state transition following S961 treatment, we first compared the β-1 normal-OxPhos state with the β-5 translation-stress state (Figure 4E and Supplemental Figure 4A). Genes upregulated in the β-5 translation-stress state were involved in translation and ER-associated processes including ribosome assembly (*Rplp0*, *Rpl6*, *Rpl8*, and *Rps27a*), protein-folding (*Calr* and *Hspa5*), peptide hormone metabolism (*Pcsk1*), and the unfolded protein response (UPR) (*Herpud1*, *Ppp1r15a*, and *Wfs1*) (Figure 4E, Supplemental Figure 4C, and Supplemental Table 2G). Genes downregulated in the β-5 translation stress state were related to β cell function, including regulation of insulin secretion, regulation of exocytosis, ion channel transport, and OxPhos (Supplemental Figure 4A). Overall, these observations highlight the increased expression of translation machinery genes and engagement of ER-associated processes as β cells adapt to hyperglycemia caused by S961.

### Sirt2 deletion alters hyperglycemia-induced shifts in the β cell transcriptional state.

Having identified β cell states associated with hyperglycemia, we asked whether SIRT2 inactivation affects glucose-induced β cell state transitions by analyzing islets from *Sirt2*^Δβ^ mice treated with S961. Indeed, the abundance of the β-3 OxPhos-ROS state was enriched at the expense of the β-5 translation-stress state in S961-treated *Sirt2*^Δβ^ islets (Figure 4, B–D). As β-3 and β-5 cells exhibited several overlapping GO term and pathway enrichments (i.e., translation and asparagine *N*-linked glycosylation), we directly compared these states by analysis of differential expression between each β cell state followed by enrichment analysis, as above. Relative to β-5 cells, the β-3 OxPhos-ROS state showed lower expression of genes involved in translation and protein processing such as genes for cytoplasmic translation, protein processing in the ER, insulin processing, and translational elongation (Supplemental Figure 4B and Supplemental Table 2H). β-3 cells exhibited differential expression of OxPhos genes in both directions compared with β-5 cells, with upregulation of nucleus-encoded components and downregulation of mitochondria-encoded components of the electron transport chain (Supplemental Figure 4, B and D). Consistent with subset-specific enrichments, β-3 cells expressed higher levels of the ROS detoxification genes *Txn1* and *Prdx1* than did β-5 cells (Supplemental Figure 4E). To analyze genes involved in the cell-type–specific stress response of the β cell, we performed gene set enrichment analysis (GSEA) using recently published transcriptional signatures of the β cell stress response (44). Ribosomal protein genes and UPR genes comprise major components of the recently described “β cell exhaustive adaptive response” observed in Min6 insulinoma cells subjected to chronic treatment of the ER stress inducer cyclopiazonic acid (CPA), which inhibits Ca^2+^ transport into the ER (44). The β cell exhaustive adaptive response has been associated with transient growth arrest in Min6 cells (44), providing a plausible link to altered proliferation. We therefore asked whether this response is activated in the above-described β cell states and found that the S961-enriched β-5 translation-stress subset indeed displayed a signature of the β cell exhaustive adaptive response, including upregulation of UPR and ribosomal protein genes, compared with the β-1 normal-OxPhos subset (Figure 4F). Conversely, β-3 OxPhos-ROS cells exhibited a much-reduced β cell exhaustive adaptive response signature compared with β-5 translation-stressed cells (Figure 4F), including reductions in signatures of both the UPR (e.g., *Herpud1*, *Hspa5*, and *Wfs1*) and of ribosomal protein genes (e.g., *Rplp0*, *Rpl6*, *Rpl8*, and *Rps27a*) (Figure 4F and Supplemental Figure 4C). As *Sirt2* deletion prevented most β cells from transitioning to the more stressed β-5 state in response to S961 treatment (Figure 4C), these observations suggest that hyperglycemia activated a stress response in β cells in a SIRT2-dependent manner. This SIRT2-dependent stress response is reminiscent of the β cell exhaustive adaptive response that includes signatures of the UPR and of ribosomal protein genes and has been described to coincide with ER stress–induced growth arrest in Min6 cells (44). Notably, we observed little effect of β cell *Sirt2* deletion on transcriptomes of other islet cell types (Supplemental Figure 3B), indicating that the effect of Sirt2 on islet cell gene expression was largely cell autonomous. Altogether, our analysis of islet metabolism and transcriptionally defined β cell states builds the working model that SIRT2 deacetylates metabolic enzymes to restrain OxPhos during S961 treatment, and this direct effect of SIRT2 on metabolism is reflected by transcriptional changes indicating differences in the stress response of β cells to hyperglycemia.

### Targeted delivery of antisense oligonucleotides against SIRT2 enhances β cell proliferation in preclinical models of increased β cell workload.

**Figure 5:**
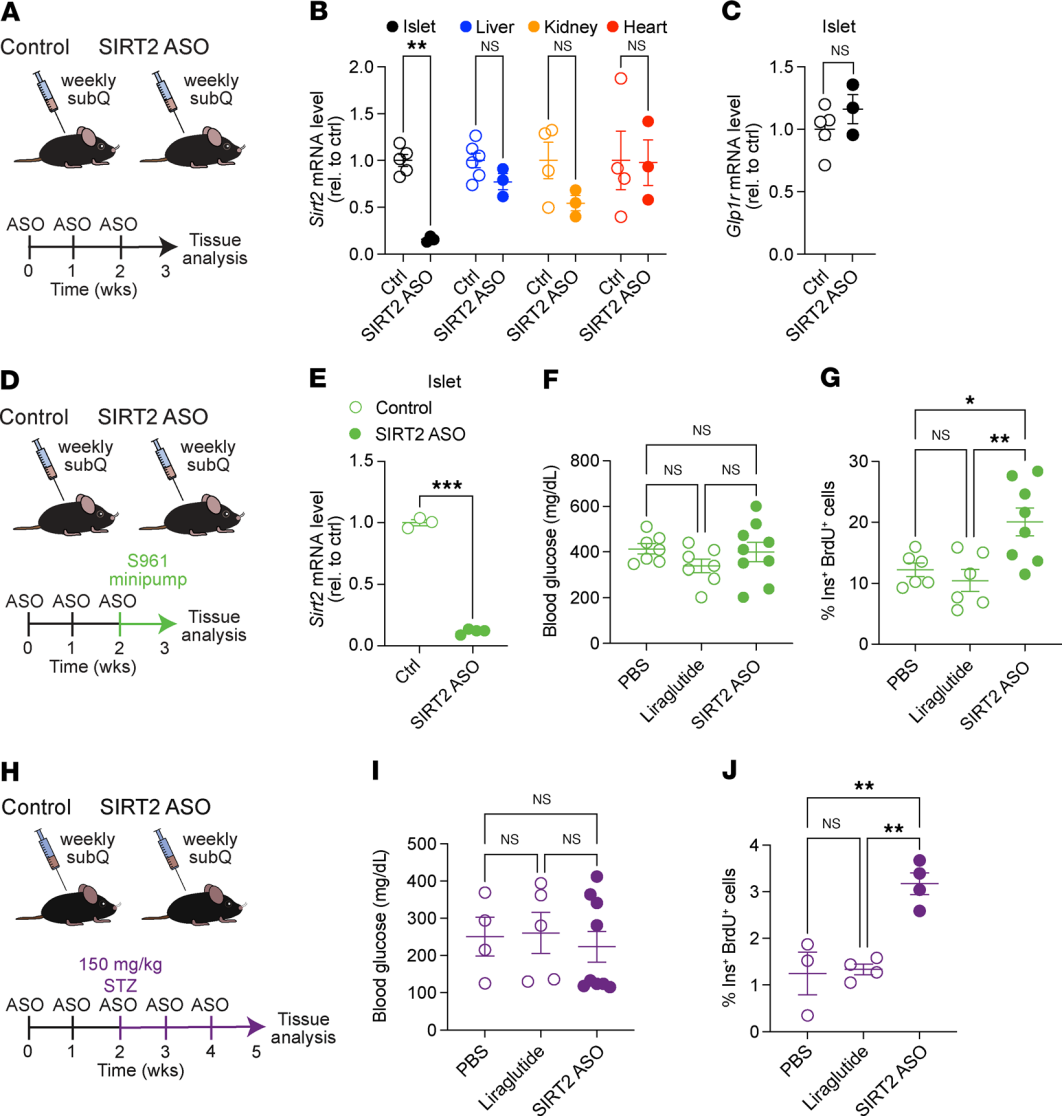
β Cell–targeted *Sirt2* ASO enhances β cell proliferation under hyperglycemic conditions in vivo. (**A**) Schematic of systemic GLP1-*Sirt2*-ASO treatment. (**B** and **C**) Quantitative PCR (qPCR) analysis of *Sirt2* mRNA levels in the indicated tissues (**B**) and *Glp1r* mRNA levels in islets (**C**). (**D**) Schematic of GLP1-*Sirt2* ASO treatment followed by implantation of S961 pumps. (**E**) qPCR analysis of *Sirt2* mRNA level in islets from S961-treated mice. (**F** and **G**) Blood glucose levels (**F**) (*n* = 7–9 mice/group) and β cell proliferation (**G**) (*n* = 6–8 mice/group) for the indicated groups of S961-treated mice. (**H**) Schematic of GLP1-*Sirt2*-ASO treatment in STZ-treated mice. (**I** and **J**) Blood glucose levels (**I**) (*n* = 4–9 mice/group) and β cell proliferation (**J**) (*n* = 3–4 mice/group) for the indicated groups of STZ-treated mice. Data are shown as the mean ± SEM. Statistical differences were calculated using a 2-way ANOVA followed by Fisher’s LSD test (**B** and **E**), unpaired, 2-tailed Student’s *t* test (**C**), or 1-way ANOVA followed by Tukey’s post hoc test (**F**, **G**, **I**, and **J**). *P < 0.05, **P < 0.01, and ***P < 0.001. rel., relative.

Considering that SIRT2 inactivation promotes β cell proliferation without interfering with insulin secretion or bypassing homeostatic set points for β cell expansion, this enzyme is an attractive therapeutic target. However, SIRT2 is expressed in non–β cells and is an important tumor suppressor in some cell types (23), underscoring the need for a high degree of cell-type selectivity were SIRT2 to be therapeutically inactivated. As cell-type–specific drug delivery remains a considerable challenge, we turned to a recently developed approach to selectively target β cells with antisense oligonucleotides (ASOs) (45). Here, an ASO targeting a gene of interest is conjugated to the peptide hormone GLP1. Following systemic administration, GLP1-conjugated ASOs are selectively internalized by GLP1R-expressing cells, enabling cell-type–specific knockdown of ASO-targeting mRNAs (45). First, we tested the effectiveness and tissue specificity of β cell–targeted *Sirt2* knockdown by systemic administration of a GLP1-conjugated ASO targeting mouse *Sirt2* mRNA (GLP1-*Sirt2* ASO) for 3 weeks. We first determined knockdown efficiency in normoglycemic mice and observed effective 84% knockdown of *Sirt2* mRNA in islets (Figure 5, A and B). We further tested knockdown in other GLP1 receptor–expressing (GLP1R-expressing) tissues (kidney, heart) as well as liver, where unconjugated ASOs have previously been shown to accumulate (46). Systemic GLP1-*Sirt2* ASO treatment had no significant effect on *Sirt2* expression in liver, kidney, or heart (Figure 5B), suggesting relative islet specificity of the GLP1-*Sirt2* ASO. Furthermore, we observed only a nonsignificant upward trend of islet *Glp1r* mRNA expression in GLP1-*Sirt2* ASO–treated mice, indicating minimal compensatory regulation of *Glp1r* expression following binding of the GLP1-conjugated ASO to GLP1R (Figure 5C). These results establish proof of principle for islet-selective *Sirt2* knockdown using GLP1-conjugated ASO.

Having validated islet-specific *Sirt2* inactivation with the GLP1-*Sirt2* ASO, we asked whether the ASO enhances β cell proliferation in preclinical models of diabetes. To confirm that metabolic stress associated with hyperglycemia does not interfere with the efficacy or selectivity of the GLP1-*Sirt2* ASO, we analyzed tissue *Sirt2* and *Glp1r* expression levels in mice that were treated with the GLP1-*Sirt2* ASO for 3 weeks and subsequently rendered acutely hyperglycemic by S961 (Figure 5D). As in normoglycemic mice, *Sirt2* knockdown was islet selective and islet *Glp1r* expression was not affected by the GLP1-*Sirt2* ASO (Figure 5E and Supplemental Figure 5, A and B). We found no effect of the GLP1-*Sirt2* ASO on blood glucose levels after S961 administration (Figure 5F) but observed a significant increase in β cell proliferation in GLP1-*Sirt2* ASO–treated mice (Figure 5G). This effect was not due to GLP1R activation, as the GLP1R agonist liraglutide did not stimulate β cell proliferation after S961 treatment (Figure 5G). Similarly, we found that the GLP1-*Sirt2* ASO increased β cell proliferation in mice rendered hyperglycemic by STZ (Figure 5, H–J). These experiments demonstrate the efficacy of β cell–targeted knockdown of *Sirt2* via ASOs in stimulating β cell proliferation under hyperglycemic conditions. Altogether, our observations indicate that targeted SIRT2 inactivation achieves controlled, glucose-dependent β cell proliferation.

## Discussion

This work identifies SIRT2 as a regulator of adaptive β cell proliferation whose inhibition increases β cell numbers while preserving feedback control of β cell mass by glucose. In mice, pharmacological or genetic inactivation of SIRT2 increases β cell proliferation in the presence of elevated glucose levels, both ex vivo and in preclinical models of diabetes, with no effect on β cell proliferation or pancreatic insulin^+^ area in euglycemic conditions. We found that SIRT2 deacetylated enzymes involved in oxidative fuel metabolism, suggesting a metabolic mechanism for the effect of SIRT2 on proliferation and distinguishing it from other drug targets proposed for therapeutic β cell expansion. Altogether, these findings establish SIRT2 as a promising therapeutic target for safely increasing β cell numbers in diabetes.

How is SIRT2 mechanistically coupled to environmental glucose concentrations in β cells? Our studies indicate several plausible mechanisms underlying the glucose dependence of SIRT2’s function. First, we found that SIRT2 deacetylated glycolytic and mitochondrial metabolic enzymes and dampened respiration in high glucose in β cells. SIRT2 deacetylation targets such as GAPDH, ACO2, and SDH are shared between β cells and T cells, where it was shown that *Sirt2* deletion leads to enzyme hyperacetylation and increased oxidative metabolism (24). As the mitogenic effect of glucose in β cells is known to involve mitochondrial metabolism, ATP synthesis, and K_ATP_ channel closure (14), we speculate that *Sirt2* deletion sensitizes β cells to adaptive proliferation through increased activity of glycolytic and TCA cycle enzymes and enhanced glucose metabolism. There are likely cell-type–specific mechanisms for metabolic regulation of proliferation by SIRT2, as glucose contributes to T cell proliferation by fueling biosynthetic reactions sustained by rapid production of lactate (47), which is minimally produced by β cells. In addition to its direct effect on metabolism, SIRT2 affects hyperglycemia-induced changes to β cell transcriptional states, providing a link between chronic elevations in glucose levels and SIRT2 function. A sustained elevation of glucose is known to activate β cell stress pathways including oxidative stress (48) as well as endoplasmic reticulum stress (49) and the corresponding UPR (50, 51). Indeed, scRNA-Seq analysis of β cells of hyperglycemic S961-treated mice identified SIRT2-dependent activation of various stress pathway signatures. Namely, *Sirt2* deletion ameliorated signatures of the UPR and of the β cell exhaustive adaptive response during hyperglycemia, indicating that SIRT2 affects how β cells interpret hyperglycemia as a stress. As stressors such as ER stress can arrest β cell proliferation (44, 49, 52), we propose that SIRT2-dependent stress responses restrain β cell expansion during hyperglycemia, thereby linking SIRT2’s effect on β cell proliferation to chronically elevated glucose. Further studies will be required to define the relative importance of altered metabolism and stress responses in the proproliferative effect of *Sirt2* inactivation, as well as the specific SIRT2 substrates mediating this effect.

The successful application of β cell mitogenic compounds as a diabetes therapy will require sufficient expansion of β cell numbers to exert a biologically meaningful effect on glycemia (53). Existing experimental drugs that show efficacy in stimulating β cell proliferation in preclinical models include those targeting the TGF-β signaling pathway (5, 54), the tumor suppressor MEN1 (9), and the kinases DYRK1A (55) and SIK1/-2/-3 (56). These compounds alter transcriptional regulation of the cell-cycle machinery either directly (5, 9, 54, 55) or, in the case of the SIK1/2/3 inhibitor HG-9-91-01, via moderate activation of the UPR (56). Combination therapy with agents exhibiting synergism has been proposed as a strategy to maximize β cell expansion (7). Here, we found that SIRT2 regulated β cell proliferation via a metabolic mechanism, opening the possibility for new combinatorial therapies utilizing distinct synergistic mechanisms.

β Cell expansion therapy in diabetes should specifically increase the proliferation of β cells without causing hyperplasia of non–β cells (53). Here, we provide proof of principle for selective β cell expansion using systemic administration of a *Sirt2*-targeting ASO conjugated to GLP1, which is selectively taken up by GLP1R-expressing cells including β cells. This targeted delivery approach is designed to reduce the risk of unintended off-target effects of inactivating SIRT2 in tissues where it functions as a tumor suppressor (23). While GLP1-conjugated ASOs against other mRNAs have shown efficacy in ameliorating β cell ER stress in diet-induced obesity (57) and β cell dysfunction in hIAPP-transgenic mice (58), to our knowledge, this work is the first to demonstrate increased β cell proliferation via this approach. Considering the potential tumorigenic effect of stimulating off-target cell proliferation, it is of particular importance that mitogenic therapies exhibit cell-type specificity. Given the here-described role of SIRT2 in restraining adaptive β cell proliferation, this work opens the possibility of therapeutically targeting SIRT2 for controlled and selective β cell expansion in diabetes.

While the here-described effects of SIRT2 inactivation on β cell proliferation are encouraging, there are some limitations to the current studies. The glucose dependence of SIRT2’s effect on β cell proliferation was different in mouse and human islets. In mice, SIRT2 inhibition increased β cell proliferation only at intermediate (8 mM) glucose concentrations. By contrast, in human islets, this effect was apparent at both basal (5 mM) and intermediate (8 mM) glucose levels. While we cannot mechanistically account for this with current experimental data, human islets are known to exhibit a left-shifted relationship between glucose concentration and utilization compared with mouse islets (59), which we speculate contributes to the species difference in the glucose concentration threshold for SIRT2’s function in β cells. Overall, as most of the studies in this work were performed in mouse models, further investigation will be necessary to determine which effects of SIRT2 inactivation (i.e., elevated islet respiration, dampening of β cell stress responses, and expansion of β cell area) are conserved in humans and the extent to which SIRT2’s function is preserved in the contexts of type 1 and type 2 diabetes. Another limitation of these studies is that we did not monitor β cell survival following SIRT2 inactivation. It remains possible that differences in the efficiency of β cell ablation by STZ contributed to differences in the pancreatic insulin^+^ area at the endpoint of the study in *Sirt2*^Δβ^ mice. Finally, we did not assess off-target effects of GLP1-*Sirt2* ASO on other GLP1R-expressing cell types (e.g., in the hypothalamus and brainstem) or at sites of passive uptake of systemically delivered ASOs (e.g., liver and kidney), nor did we comprehensively test indirect effects of β cell SIRT2 inactivation on other islet cell types. Further mechanistic studies of SIRT2 in β cells, validation of SIRT2’s specific functions in humans, and comprehensive examination of off-target effects will be important for assessing the feasibility of expanding β cells through the here-described therapeutic strategy.

## Methods

### Sex as a biological variable.

Both male and female mice were used for all experiments. Sex was not considered as a biological variable.

### Animal studies.

The following mouse strains were used in this study: *C57BL/6* (Charles River Laboratories), *Sirt2^fl^* (60), *Pdx1-CreER* (61), and *MIP-CreER* (62). β Cell–specific *Sirt2* deletion was generated by crossing *Sirt2^fl^* mice with *Pdx1-CreER* or *MIP-CreER* mice that were subcutaneously injected with tamoxifen (MilliporeSigma), 4 doses of 6 mg every other day, at in 12–15 weeks of age. Control *Sirt2^+/+^* or *Sirt2^fl/+^ CreER* mice were injected with tamoxifen in parallel. Post-tamoxifen time points are expressed relative to the final tamoxifen injection. Mice were weaned at 4 weeks, maintained on a 12-hour light cycle, and fed standard rodent chow (PicoLab Rodent Diet, 20 5053) ad libitum. To render mice hyperglycemic, 12- to 15-week-old mice were treated with either STZ or S961 starting 3 weeks after the final tamoxifen injection. For the STZ experiment, mice were fasted for 4 hours before receiving an intraperitoneal injection of STZ (MilliporeSigma) dissolved in citrate buffer (pH 4.5) at doses of 150 or 200 mg/kg body weight for GLP1-*Sirt2*-ASO studies and genetic *Sirt2* inactivation studies, respectively. Animals were given 10% sucrose water overnight. The insulin receptor antagonist S961 (Celtek Peptides) was administrated at 20 nmol/week with an osmotic minipumps (Alzet 1007D) for 1 week. To target *Sirt2* in β cells, 12- to 15-week-old *C57BL/6* mice were subcutaneously injected once weekly with 1 mg/kg GLP1-conjugated ASO (45, 63) against *Sirt2* (sequence with cEt chemistry wings underlined: GTAAGATACTGCACAA) or a control (GGCCAATACGCCGTCA) for 3 weeks before hyperglycemia was induced via S961 or STZ as described above. Body weight and blood glucose levels were measured weekly using a glucometer (Bayer Contour glucometer; Bayer). For BrdU labeling, mice were given 0.8 mg/mL BrdU in drinking water for 7 days prior to harvesting pancreata.

### Islet isolation and islet culture.

Mouse islet isolation was performed by collagenase digestion of the adult pancreas as previously described (21). Briefly, mouse pancreata were perfused through the common bile duct with Collagenase P (MilliporeSigma), and islets were purified by density-gradient centrifugation using Histopaque 1.077 (MilliporeSigma). Islets were allowed to recover from the isolation and cultured in RPMI 1640 medium (CellGro) supplemented with 10% FBS, 8 mM glucose, 2 mM glutamine (Corning), 100 U/mL penicillin/streptomycin (Gibco, Thermo Fisher Scientific), 1 mM sodium pyruvate (CellGro), 10 mM HEPES (Gibco, Thermo Fisher Scientific), and 0.25 mg/mL amphoterecin B at 37°C with 5% CO_2_ for 12–48 hours.

Human islets were received through the Integrated Islet Distribution Program (IIDP). Upon receipt, islets were stained with 0.02 μg/mL dithizone and 0.1 mM ammonium hydroxide solution for 10 minutes at 37°C, and hand-selected islets were cultured in CMRL 1066 (CellGro) supplemented with 10% FBS, 1.22 μg/mL nicotinamide, 1:1,000 insulin-selenium-transferrin (Gibco, Thermo Fisher Scientific), 16.7 μM zinc sulfate, 5 mM sodium pyruvate (CellGro), 2 mM GlutaMAX (Gibco, Thermo Fisher Scientific), 25 mM HEPES (Gibco, Thermo Fisher Scientific), and 100 U/mL penicillin/streptomycin (Gibco, Thermo Fisher Scientific). Detailed human islet donor information is listed in Supplemental Table 3.

Overnight-recovered mouse or human islets were cultured with 0.1% DMSO (vehicle) or 9 μM AGK2 (Tocris), 25 μM AK-1 (MilliporeSigma), 10 μM SirReal2 (Tocris), 100 μM NMN (MilliporeSigma), or combinations of the above as indicated under various glucose concentrations (5 mM, 8 mM, 16.8 mM). To detect proliferating cells, islet culture media were supplemented with 10 μM EdU. After 48 hours, islets were fixed in 4% paraformaldehyde and stained as described below.

### Immunohistochemical analysis.

Mouse pancreata were fixed in 4% paraformaldehyde at 4°C overnight. Human and mouse islet samples were fixed in 4% paraformaldehyde at room temperature for 30 minutes. After fixation, samples were washed 3 times with PBS and then incubated in 30% sucrose at 4°C overnight. Pancreata and islet samples were embedded with Optimal Cutting Temperature Compound (Tissue-Tek), frozen in a 100% ethanol/dry-ice bath, and sectioned at 10 μm using a Cryostat (Leica).

Mouse pancreata or human and mouse islet sections were then washed with PBS for 5 minutes and permeabilized and blocked with 1% normal donkey serum and 0.15% Triton X-100 (Thermo Fisher Scientific) in PBS for 1 hour. For detection of BrdU, antigen retrieval was performed by treatment with 2 M hydrochloric acid for 30 minutes followed by 0.1 M sodium borate for 5 minutes at room temperature prior to permeabilization and blocking. The following primary antibodies were used: guinea pig anti-insulin (Dako, A0564, 1:1,000), rat anti-BrdU (Novus Biologicals, NB500-169, 1:250), rabbit anti-Pdx1 (Abcam, AB47267, 1:500), rabbit anti-Nkx6.1 (LifeSpan BioSciences, LS-C143534, 1:250), goat anti-glucagon (Santa Cruz Biotechnology, SC-7780, 1:1,000), and rabbit anti-H2AX (Cell Signaling Technology, 2577, 1:100). Dilutions were prepared in blocking solutions and sections were incubated overnight at 4°C. After washing, the sections were incubated with donkey-raised secondary antibodies conjugated to Alexa Fluor 488, Cy3, or Cy5 (Jackson ImmunoResearch) for 1 hour at room temperature. Nuclei were counterstained with DAPI (MilliporeSigma) at 0.1 μg/mL. EdU was detected using the Click-iT EdU Alexa Fluor 488 kit as specified by the manufacturer (Life Technologies, Thermo Fisher Scientific).

Images were captured on a Zeiss Axio Observer Z1 microscope with an ApoTome module and processed with Zeiss AxioVision 4.8 software. Images were analyzed using HALO software (Indica Labs). Proliferation of β cells was quantified as a percentage of insulin^+^ and EdU^+^ or BrdU^+^ cells relative to total cell numbers. For β cell area measurements, images covering an entire pancreas section were tiled using a Zeiss Axio Observer Z1 microscope with the Zeiss ApoTome module. The insulin^+^ and total pancreas areas were measured using ImageJ (NIH), and the relative β cell area was calculated as the insulin^+^ area relative to the total pancreas area.

### Insulin secretion measurements.

Static insulin secretion assays were performed essentially as described previously (21) after islets were allowed to recover overnight from isolation (mouse islets) or shipment (human islets). Islets were then preincubated for 1 hour at 37°C with CO_2_ in Krebs-Ringer bicarbonate HEPES (KRBH) buffer (130 mM NaCl, 5 mM KCl, 1.2 mM CaCl_2_, 1.2 mM MgCl_2_, 1.2 mM KH_2_PO_4_, 20 mM HEPES pH 7.4, 25 mM NaHCO_3_, and 0.1% BSA) containing 2.8 mM glucose. Afterwards, islets were size matched and groups of 10 islets were transferred to a 96-well dish into KRBH solutions of 2.8 mM glucose or 16.8 mM glucose. After incubation for 1 hour as above, supernatant was collected, and islets were lysed overnight in a 2% HCl/80% ethanol solution. Secreted insulin and islet insulin content were determined using a mouse or human insulin ELISA kit (ALPCO). Secreted insulin was calculated as a percentage of total insulin content.

### Metabolic studies.

For glucose tolerance tests (GTTs), fasted mice (6 hours) were injected intraperitoneally with a dextrose solution at a dose of 1.5 g/kg body weight. Plasma glucose levels were measured in blood samples from the tail vein at baseline, 20 minutes, 40 minutes, 60 minutes, 90 minutes, 120 minutes, and 150 minutes after glucose injection using a Bayer Contour glucometer (Bayer).

### Western blotting.

Islets were lysed in RIPA buffer containing protease inhibitor cocktail (Roche) and phosphatase inhibitor (MilliporeSigma). Protein concentrations were determined by BCA assay (Thermo Fisher Scientific). Lysates (30 μg) were analyzed by SDS-PAGE on 12% Bis-Tris gels. Proteins were visualized after transfer onto a PVDF blotting membrane (GE Healthcare) and incubation with specific primary antibodies using HRP-conjugated secondary antibodies. Western blot primary antibodies included rabbit anti-Sirt2 (Santa Cruz Biotechnology, SC-20966, 1:250) and mouse anti-tubulin (MilliporeSigma T5201, 1:1,000) antibodies. Western blot secondary antibodies included donkey anti-rabbit HRP (VWR 95017, 1:1,000) and sheep anti-mouse (GE Healthcare, NA931V, 1:1,000) antibodies. Protein bands were detected using Chemiluminescent Substrate (Thermo Fisher Scientific) on Blue Bio Films (Denville Scientific) with an SRX-101A developer (Konica Minolta). Densitometric analysis was performed using ImageJ.

### Acetylome analysis.

Human islets from 6 donors (*n* = 8,000 islets/donor and condition) were cultured in 8 mM glucose with either the SIRT2 inhibitor AGK2 or DMSO control as described above, and islets were then pelleted and stored at –80°C. Samples were pooled in 200 μL of 6 M guanidine-HCl and then subjected to 3 cycles of 10 minutes of boiling and 5 minutes of cooling at room temperature. The proteins were precipitated with addition of methanol to a final volume of 90% followed by vortex and centrifugation at 13,090*g* on a benchtop microfuge for 10 minutes. The soluble fraction was removed by flipping the tube onto an absorbent surface and tapping to remove any liquid. The pellet was suspended in 200 μL of 8 M urea, 100 mM Tris pH 8.0. Next, Tris-(2-carboxyethyl) phosphine hydrochloride (10 mM) and chloro-acetamide (40 mM) were added, and the samples were then vortexed for 5 minutes. Three volumes of 50 mM Tris pH 8.0 and a 1:50 ratio of trypsin were added and samples were incubated at 37°C for 12 hours. Samples were then acidified with 0.5% trifluoroacetic acid and then desalted using Phenomenex Strata-X 33 μm solid-phase extraction columns (part no. 8B-S100_UBJ) as described by the manufacturer’s protocol. The peptides were dried by speed-vac and then resuspended in 1 mL PBS. Fifty micrograms of each anti–acetyl-lysine (acetyl-lys) antibody-bead conjugate (AKL5C1, Santa Cruz Biotechnology and ICP0388, ImmuneChem) was added to the solution and mixed for 1 hour. After 3 washes with PBS, the peptides were eluted using 1% TFA solution. Seventy-five percent of the enriched fraction was analyzed by ultra-high-pressure liquid chromatography (UPLC) coupled with tandem mass spectroscopy (LC-MS/MS) using nanospray ionization. The nanospray ionization experiments were performed using an Orbitrap fusion Lumos hybrid mass spectrometer (Thermo Fisher Scientific) interfaced with nanoscale reversed-phase UPLC (Thermo Dionex UltiMate 3000 RSLC Nano System) using a 25 cm, 75 μm ID glass capillary packed with 1.7 μm C18 (130) Ethylene Bridged Hybrid beads (Waters Corporation). Peptides were eluted from the C18 column into the mass spectrometer using a linear gradient (5%–80%) of acetonitrile with 0.1% formic acid at a flow rate of 375 μL/min for 180 minutes. The mass spectrometric parameters were as follows: an MS1 survey scan using the orbitrap detector (mass range [*m/z*]: 400–1,500, using quadrupole isolation, a 60,000 resolution setting, a spray voltage of 2,200 V, an ion transfer tube temperature of 275°C, an AGC target of 400,000, and a maximum injection time of 50 ms) was followed by data-dependent scans (top speed for most intense ions, with the charge state set to only include +2–5 ions, and a 5 second exclusion time, while selecting ions with minimal intensities of 50,000, at which the collision event was carried out in the high-energy collision cell [HCD collision energy of 30%]), and the fragment masses were analyzed in the ion trap mass analyzer (ion trap scan rate of turbo, first mass *m/z* = 100, AGC target = 5,000, and maximum injection time = 35 ms). Protein identification was carried out using Peaks Studio 8.5 (Bioinformatics Solutions). Proteins identified as being acetylated that also exhibited 1.5-fold or higher differences in abundance between AGK2- and DMSO-treated human islet acetyl-lys fractions (Supplemental Table 1) were considered differentially acetylated.

### Islet respirometry.

Respirometry was performed using a protocol adapted from ref. 64. Briefly, islets were starved in Seahorse XF DMEM Assay Medium supplemented with 1% FBS, 2.8 mM glucose, and 5 mM HEPES (final pH of 7.4) at 37°C without CO_2_ for 1 hour. Following starvation, the islets were seeded at a density of 10 size-matched islets per well into Matrigel-coated XFe96 Cell Culture Microplates containing Seahorse XF DMEM Assay Medium supplemented as above. The plate was then loaded into the Seahorse Bioscience XF96 Extracellular Flux Analyzer heated to 37°C, and oxygen consumption was measured sequentially for the following states: basal (2.8 mM glucose), glucose-stimulated (16.8 mM glucose), ATP synthase–independent (5 mM oligomycin), and nonmitochondrial (2 μM antimycin and 0.5 μM rotenone) respiration. Maximum uncoupled respiration was measured by treating with 1.5 μM carbonyl cyanide 4-(trifluoromethoxy)phenylhydrazone (FCCP) after the addition of oligomycin. Following respirometry, wells were imaged, and the relative islet area was calculated using ZEN software. Oxygen consumption rates (OCRs) for each well were normalized to the islet area relative to that of all wells. For normoglycemic mice, islets were cultured in standard islet medium (as described above) containing 8 mM glucose for 48 hours before performing islet respirometry. Respirometry experiments were performed the same day as islet isolation for islets isolated from S961-treated mice.

### scRNA-Seq data preprocessing, quality control, and analysis.

The 10x Genomics CellRanger analysis pipeline (“cellranger count,” version 2.1.0) was used for: (a) aligning the reads from the demultiplexed FASTQ files to the mm10 reference genome build (10x Genomics); (b) filtering, and (c) barcode and unique molecular identifier (UMI) counting, to generate gene expression matrices. The cell-calling algorithm in CellRanger automatically excludes barcodes that correspond to background noise, yielding a filtered gene expression matrix. The individual count matrices were further aggregated into a single-feature barcode matrix using the “cellranger aggr” function. All further downstream analysis, unless otherwise stated, was performed with R 4.0.4.

The aggregated feature-barcode matrix was imported and converted into a SeuratObject using the “CreateSeuratObject” function provided by the Seurat (version 3) package. Data transformation and normalization were performed using SCTransform to fit a negative binomial distribution and regress out mitochondrial read percentage. Principal components (PCs) were then calculated and used downstream for unsupervised clustering and dimensionality reduction. Clustering was performed using “FindNeighbors” with the first 30 PCs, and the UMAP dimension reduction was then computed on the same 30 PCs using the “RunUMAP” function.

After initial clustering analysis, cells were filtered for mitochondrial reads below 5% and 1,000 < nFeature_RNA < 5,000. Polyhormonal cells were defined by setting hormone-specific thresholds for the 4 islet endocrine cell types (α: *Gcg*; β: *Ins1*; δ: *Sst*; γ: *Ppy*). On the basis of this, polyhormonal doublets and endocrine-nonendocrine doublets were excluded from downstream analysis. PCs, neighbors, and UMAP dimensional reduction were then recomputed for the remaining cells. Based on the new PCs and neighbors, *k*-means clustering was performed, and the resulting clusters were annotated on the basis of hormone expression.

The clusters annotated as “Beta” were subset from the full, aggregated data and renormalized and recomputed to obtain a β-specific “SeuratObject.” For this β object, new subsets were computed at a resolution of 0.3 using the “FindClusters” function, yielding 5 subsets (β-1 through β-5). The subset markers were identified using the “FindAllMarkers” function with default parameters and were subsequently used to identify functional categories from the Kyoto Encyclopedia of Genes and Genomes (KEGG) and Reactome categories with Metascape.

### RNA analysis.

Total RNA was isolated from islets or homogenized tissues and purified using RNeasy columns and RNase-free DNase digestion according to the manufacturer’s instructions (QIAGEN). The quality and quantity of total RNA were monitored and measured with NanoDrop (NanoDrop Technologies) following the manufacturer’s instructions. For quantitative reverse transcription PCR (RT-qPCR) analysis, cDNA was synthesized using the iScript cDNA Synthesis Kit (Bio-Rad) and 500 ng isolated RNA per reaction. Template cDNA (20 ng per reaction) and iQ SYBR Green Supermix (Bio-Rad) were used for real-time PCR with gene-specific primers (Supplemental Table 4), and *Tbp* was used as a housekeeping gene on a CFX96 Real-Time PCR Detection System (Bio-Rad).

### Statistics.

Statistical analyses were performed using GraphPad Prism 9 (GraphPad Software). Normality was tested with a Shapiro-Wilk test, and *F* tests were performed to analyze equal variances. Data that passed both tests were analyzed by 2-tailed Student’s *t* test for 2-group comparisons and 1-way ANOVA for comparison of multiple groups (>2) followed by Tukey’s post hoc testing. For data with multiple variables, e.g. glucose measurements over time, a 2-way ANOVA for repeated measurements followed by Tukey’s post hoc test or Fisher’s least significant difference post hoc testing was performed. All data are presented as the mean ± SEM. *P* values of less than 0.05 were considered significant.

### Study approval.

All mice were housed and bred in the UCSD School of Medicine vivaria approved by the Association for Assessment and Accreditation of Laboratory Animal Care, following standards and procedures approved by the UCSD IACUC.

### Data availability.

scRNA-Seq data were deposited in the NCBI’s Gene Expression Omnibus (GEO) database (GSE252859). Values for all data points in graphs are provided in the Supporting Data Values file.

## Author contributions

MW and BR designed research studies, conducted experiments, acquired data, analyzed data, and wrote the manuscript. CZ and JRB designed research studies, conducted experiments, acquired data, and analyzed data. DPP, FL, M Schlichting, ARH, BL, TPP, SB, and STL conducted experiments and acquired data. SS, ECP, HZ, VAR, and KLW analyzed data. JA provided key reagents. OSS designed research studies. M Sander designed research studies, analyzed data, and wrote the manuscript. The order of the co–first authors’ names was assigned on the basis of the relative contributions to data generation, interpretation of results, and writing of the manuscript.

## Supplementary Material

Supplemental data

Unedited blot and gel images

Supplemental table 1

Supplemental table 2

Supporting data values

## Version 1 07/31/2025

In-Press Preview

## Version 2 10/01/2025

Electronic publication
