# Supplementary material for: The protein deacetylase SIRT2 exerts metabolic control over adaptive β cell proliferation
Source: J Clin Invest. 2025 Jul 31;135(19):e187020. doi: 10.1172/JCI187020 (PMC12483614; doi:10.1172/JCI187020)
Supplement: Supplemental data [file jci-135-187020-s237.pdf]

Supplemental Information

Supplemental Figures

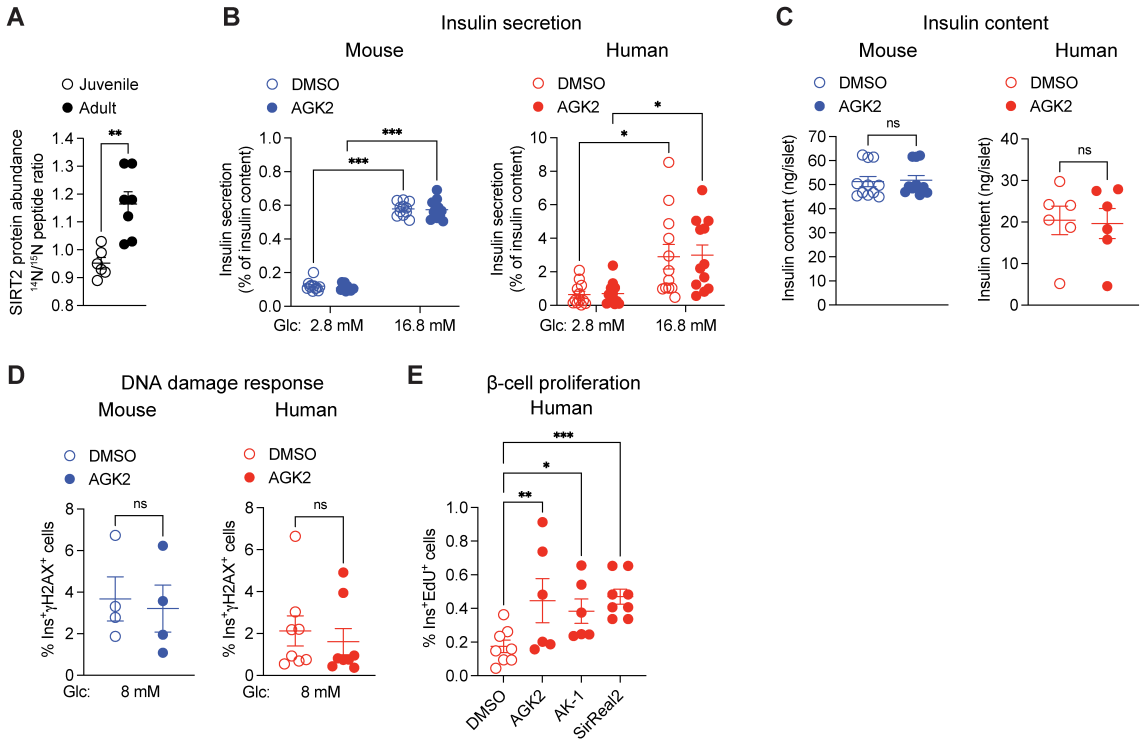

Supplemental Figure 1. SIRT2 inhibition does not affect β-cell function.
(**A**) Relative protein abundance for SIRT2 in islets from juvenile (one-month-old) and adult (one-year-old) mice (*n* = 4 pools of islets from distinct biological replicates/group with each peptide of SIRT2 shown as an individual data point).
(**B** and **C**) Glucose stimulated insulin secretion (B) and insulin content (C) were measured in isolated mouse (blue, *n* = 11 islet preparations/group) and human (red, B: *n* = 12, C: *n* = 6 islet preparations/group) islets after DMSO or AGK2 treatment in the indicated glucose concentrations.
(**D**) Quantification of γH2AX-positive β-cells in isolated mouse (blue, *n* = 4 islet preparations/group) and human (red, *n* = 8 islet preparations/group) islets after DMSO or AGK2 treatment in 8 mM glucose.
(**E**) Quantification of β-cell proliferation as a percentage of insulin-positive and EdU-positive cells relative to total β-cell numbers in human islets (red, *n* = 6-8 islet preparations/group) after treatment with DMSO, AGK2, AK-1, and SirReal2 in 8 mM glucose.
Data are shown as mean ± SEM. Statistical differences were calculated using unpaired t-test (A), two-way ANOVA with Tukey post hoc analysis (B), paired t-test (C, D), or one-way ANOVA with Tukey post hoc analysis (E). *$p<0.05$, **$p<0.01$, ***$p<0.001$; ns, not significant.

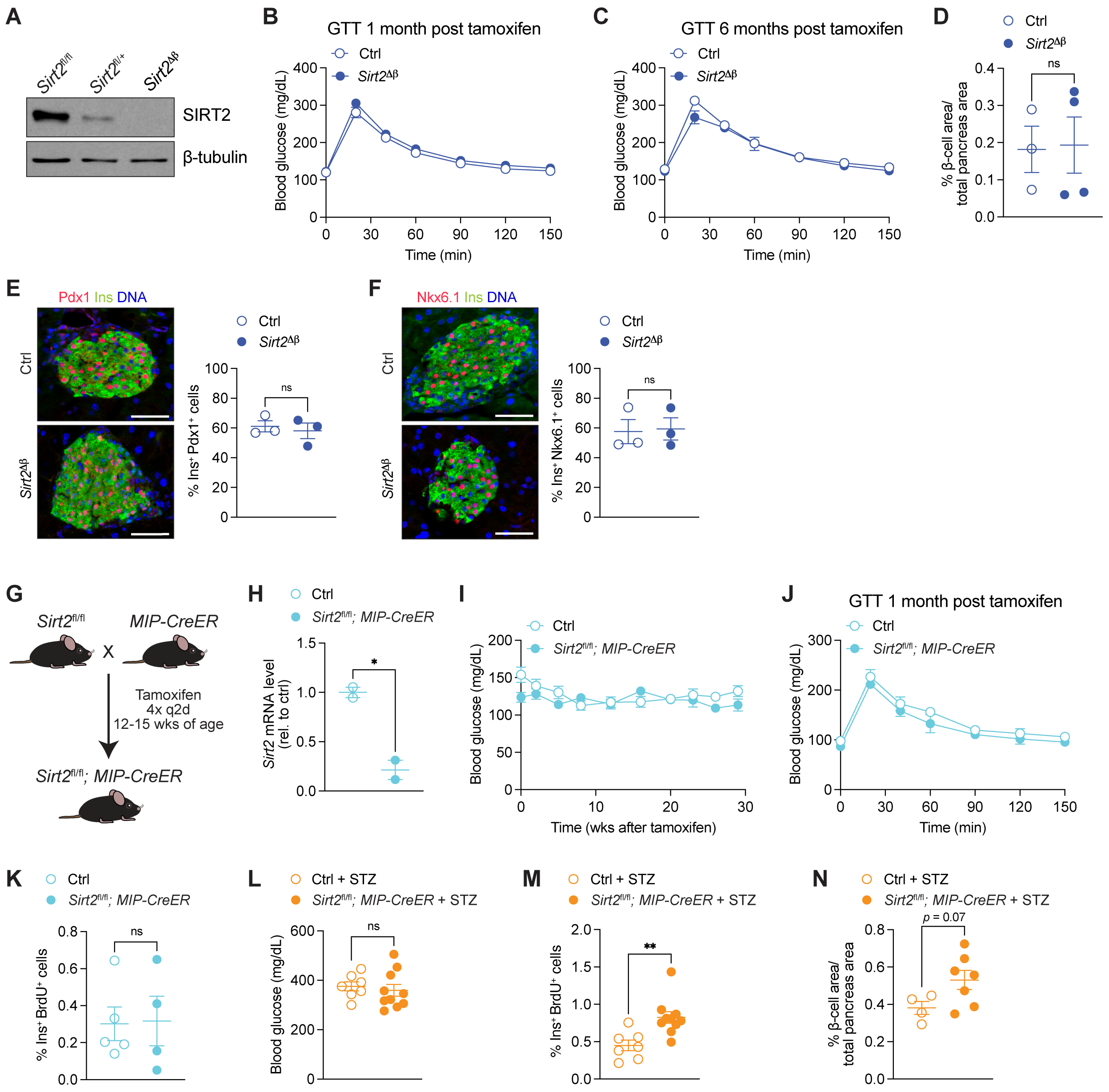

Supplemental Figure 2. Effect of *Sirt2* deletion on glucose homeostasis and β-cell proliferation.

(**A**) Immunoblot analysis of SIRT2 and β-tubulin in mouse islets of control or *Sirt2*$^{\Delta\beta}$ mice. Islets were pooled from 3 mice/genotype.

(**B** and **C**) Blood glucose levels at indicated time points after an intraperitoneal glucose injection 1 month (B) and 6 months (C) after tamoxifen treatment (n = 5-9 mice/group). Tamoxifen-treated *Sirt2*$^{+/+}$; *Pdx1CreER* and *Sirt2*$^{fl/+}$; *Pdx1CreER* mice were used as controls.

(**D**) Quantification of β-cell area relative to whole pancreas area (n = 3-4 mice/group).

(**E** and **F**) Left, representative immunofluorescence staining for the indicated proteins. Right, quantification of the percentage of β-cells expressing Pdx1 (E) or Nkx6.1 (F) for the indicated genotypes 4-6 weeks following tamoxifen treatment. DAPI was used for DNA counterstain. Scalebar is 50 μm. n = 3 mice per genotype.

(**G**) Schematic for generating β-cell-specific *Sirt2* deficient mice. Tamoxifen-treated *Sirt2*$^{+/+}$; *MIPCreER* and *Sirt2*$^{fl/+}$; *MIPCreER* mice were used as controls.
(**H**) qPCR analysis of *Sirt2* mRNA level in islets from β-cell-specific *Sirt2* deficient mice relative to control mice (n = 2 mice/group).
(**I**) Blood glucose levels measured for 30 weeks following tamoxifen treatment (n = 11 mice/group).
(**J**) Blood glucose levels at indicated time points after an intraperitoneal glucose injection (n = 4-8 mice/group).
(**K**) Quantification of β-cell proliferation as percentage of insulin-positive and BrdU-positive cells relative to total β-cell numbers 4-6 weeks post tamoxifen treatment (n = 4-5 mice/group).
(**L-N**) Hyperglycemia was induced in control and β-cell-specific *Sirt2* deficient mice by intraperitoneal injection on STZ (200 mg/kg body weight). After 3 weeks, blood glucose levels (L; n = 7-10 mice/group), β-cell proliferation (M; n = 7-10 mice/group) and β-cell area (N; n = 4-7 mice/group) were measured.
Data are shown as mean ± SEM. Statistical differences were calculated using a two-way ANOVA with Tukey post hoc analysis (B, C, I, J) or an unpaired t-test (D-F, H, K-N). *p<0.05, **p<0.01; ns, not significant. q2d, every 2 days; wks, weeks.

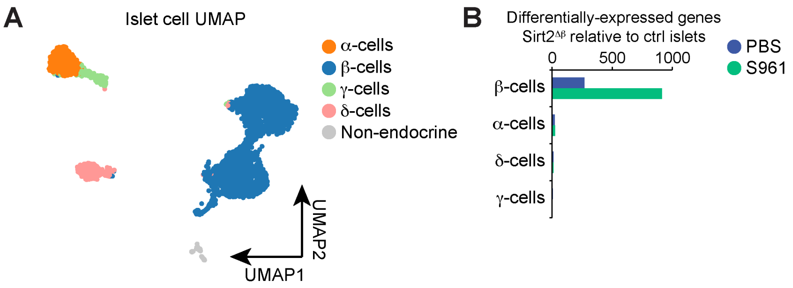

Supplemental Figure 3. Effect of β-cell *Sirt2* deletion upon transcriptomes of other islet cell types.

(**A**) UMAP (uniform manifold approximation and projection) plot of all islet cells colored by cell type.

(**B**) Bar plot indicating numbers of differentially expressed genes (FDR < 0.05) between control and $Sirt2^{\Delta\beta}$ islet cells for each cell type in the indicated conditions. FDR was determined by Wilcoxon rank-sum test. Ctrl, control.

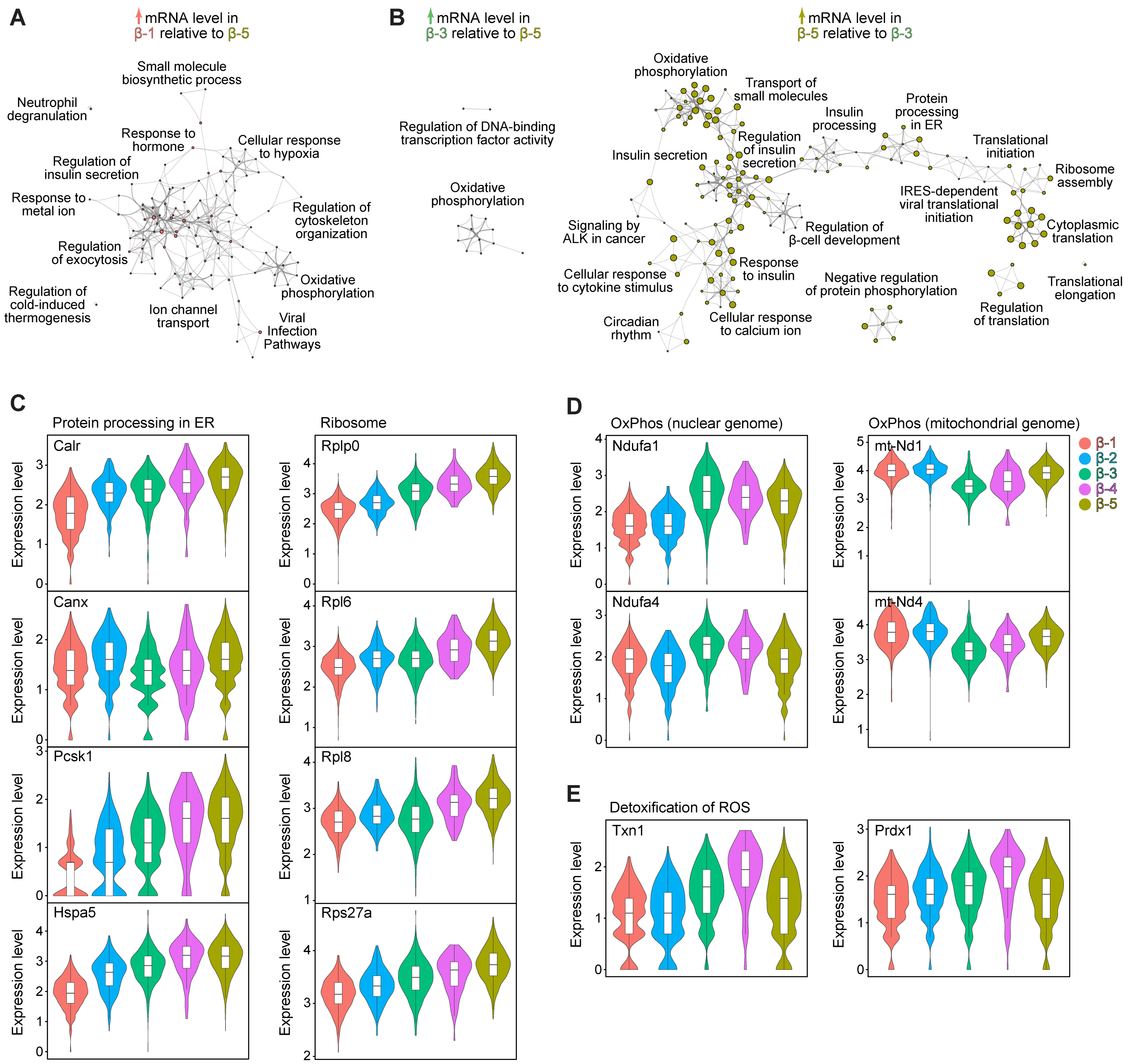

Supplemental Figure 4. Transcriptional differences between β-cell states.
(**A** and **B**) Networks of gene ontologies and pathways for mRNAs more highly expressed in β-1 compared to β-5 cells (A) and for mRNAs more highly expressed for the indicated comparisons (B). FDR < 0.05.
(**C-E**) Violin plots of the indicated mRNAs for each β-cell subset.

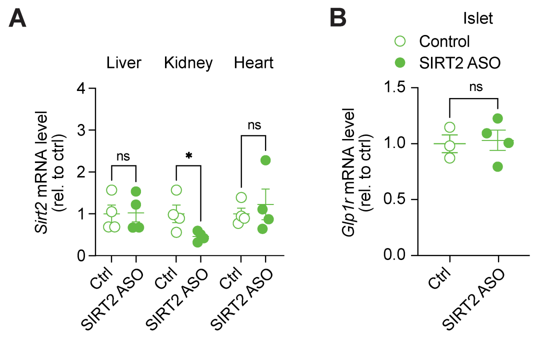

Supplemental Figure 5. Selectivity of *Sirt2* knockdown by GLP1-*Sirt2*-ASO followed by S961 treatment.

(**A** and **B**) qPCR analysis of *Sirt2* (A) and *Glp1r* (B) mRNA levels for the indicated tissues from S961-treated mice.

Data are shown as mean ± SEM. Statistical differences were calculated using two-way ANOVA followed by Fisher's LSD test (A) or unpaired t-test (B). **p*<0.05, ns, not significant.

Supplemental Tables

Supplemental Table 1. Acetylome analysis in human islets.
(A) All detected peptides and corresponding fold changes in intensity between AGK2- and DMSO-treated human islet lysates enriched for acetyl-Lys.
(B) Peptides identified as being acetylated from (A).
(C) GO term and pathway enrichment analysis of proteins whose corresponding peptide fragments were identified as being acetylated that also exhibited $\geq$ 1.5-fold differences in abundance between AGK2- and DMSO-treated human islet acetyl-Lys fractions.
(Supplied as Excel file: Supplementary_Table_1.xlsx)

Supplemental Table 2. Single cell RNA-seq analysis of control and *Sirt2*$^{\Delta\beta}$ mice treated with PBS or S961.
(A) mRNAs enriched in the indicated β-cell subsets relative to all other β-cells.
(B-F) GO term and pathway enrichment analysis of mRNAs highly expressed in each subset relative to all other β-cells (from A) for mRNAs enriched in β-1 (B), β-2 (C), β-3 (D), β-4 (E), and β-5 cells (F).
(G and **H)** Pairwise differential expression analysis of β-cell subsets comparing β-1 normal-OxPhos cells with β-5 translation-stress cells (G) or comparing β-3 OxPhos-ROS cells with β-5 translation-stress cells (H).
(Supplied as Excel file: Supplementary_Table_2.xlsx)

Supplemental Table 3. Human islet donor information.
Donor information, isolation center, and data generated for each human islet prep. All tissue was obtained through the Integrated Islet Distribution Program. All donors were nondiabetic.

Human islet donor information (proliferation and GSIS experiments)

RRID:	SAMN08783909	SAMN08783902	SAMN08776506	SAMN08776503	SAMN08775096	SAMN08775090	SAMN08775023	SAMN08775047	SAMN08774967	SAMN08774953	SAMN08774820
Age	53	50	40	56	47	32	61	52	46	29	23
Sex	M	F	M	M	F	M	F	M	F	M	F
BMI	27.3	27.8	35.4	23.8	22.5	23.1	30.8	36.7	20.3	32.6	24.7
HbA1c	ND	ND	ND	ND	ND	ND	ND	ND	ND	ND	ND
Cause of death	CV/stroke	CV/stroke	Head trauma	Head trauma	CV/stroke	Anoxia	CV/stroke	CV/stroke	CV/stroke	Head trauma	Head trauma
Source	Prodo	UPenn	UPenn	COH	UPenn	Prodo	Prodo	UPenn	Miami	Miami	Prodo
Use in study	AGK2 prolif	AGK2 prolif	AGK2 prolif	AGK2 prolif	AGK2 prolif	AGK2 prolif	AGK2 prolif	AGK2 prolif	AGK2 prolif	AGK2 prolif	AGK2, AK1/ SR2, prolif, γH2AX

Human islet donor information, continued (proliferation and GSIS experiments)

RRID:	SAMN08774480	SAMN08774472	SAMN08774194	SAMN08784507	SAMN08773854	SAMN08930712	SAMN08773781	SAMN08769836	SAMN08769806	SAMN08769393	SAMN08769390
Age	24	35	68	30	49	26	42	56	36	53	33
Sex	F	M	M	F	M	M	F	F	M	F	F
BMI	35.4	32.9	26.7	18.4	40.1	44.8	23.2	33.5	28.5	32.0	34.2
HbA1c	ND	5.6	5.3	ND	5.4	4.7	5.4	5.2	5.6	5.7	5.5
Cause of death	CV/stroke	Head trauma	Head trauma	Anoxia	Anoxia	Head trauma	CV/stroke	Head trauma	Head trauma	CV/stroke	CV/stroke
Source	Miami	Miami	Prodo	UPenn	COH	Illinois	UPenn	Prodo	Miami	Prodo	Wisconsin
Use in study	AGK2, AK1/SR2 prolif, γH2AX	AGK2, AK1/SR2 prolif, γH2AX	AGK2, AK1/SR2 prolif, γH2AX	AGK2, AK1/SR2, NMN, non β-cell prolif, γH2AX	AGK2 prolif	AGK2, NMN, non β-cell prolif, γH2AX, GSIS	AGK2, AK1/SR2, NMN, non β-cell prolif, γH2AX, GSIS	GSIS	AGK2 prolif, GSIS	GSIS	GSIS

Human islet donor information, continued (proliferation and GSIS experiments)

RRID:	SAMN08769206	SAMN08769132	SAMN08768969	SAMN08769031	SAMN08769028	UNOS AEK1071	SAMN08743022	SAMN09091256	SAMN09393858	SAMN44571547	SAMN44779828
Age	52	23	33	62	63	59	33	45	43	49	58
Sex	F	M	M	M	F	M	F	M	F	M	F
BMI	25.8	24.8	30.9	28.9	20.0	27.2	32.3	29.3	34.3	24.7	26.3
HbA1c	5.6	5.3	5.7	5.5	5.0	5.1	4.9	5.0	4.6	5.0	5.5
Cause of death	CV/stroke	Anoxia	Head trauma	CV/stroke	CV/stroke	CV/stroke	Anoxia	CV/stroke	CV/stroke	CV/stroke	CV/stroke
Source	Miami	Prodo	Prodo	COH	Miami	COH	Prodo	Prodo	Prodo	Prodo	Wisconsin
Use in study	GSIS	AK1/ SR2, prolif, GSIS	AGK2, AGK2+/- NMN prolif	AGK2, AGK2+/- NMN prolif	AGK2, AGK2+/- NMN prolif	AGK2, AGK2+/- NMN prolif	AGK2, AGK2+/- NMN prolif	AGK2 prolif, non β-cell prolif	AGK2, AGK2+/- NMN prolif, non β-cell prolif	AGK2 prolif	AGK2 prolif

Human islet donor information, continued (proliferation and GSIS experiments)

RRID:	SAMN45149991	SAMN46223636
Age	66	57
Sex	M	M
BMI	32	30.5
HbA1c	5.6	5.3
Cause of death	Anoxia	CV/stroke
Source	COH	Prodo
Use in study	AGK2 prolif	AGK2 prolif

ND, not determined; CV, cardiovascular; Prodo, Prodo Labs Human Islet Isolation Center; Illinois, University of Illinois; Miami, University of Miami; UPenn, University of Pennsylvania; COH, Southern California Islet Cell Resource Center at City of Hope; Wisconsin, University of Wisconsin Human Islet Core; SR2, SirReal2.

Human islet donor information, continued (acetyl-Lys study)

RRID:	SAMN13050553	SAMN13049263	SAMN13570019	SAMN13739565	SAMN13836615	SAMN13972304
Age	25	38	37	42	58	49
Sex	M	M	F	M	M	M
BMI	29.3	24.5	24.0	37.4	23.3	34.8
HbA1c	5.3	5.3	5.2	5.6	5.7	5.5
Cause of death	Head trauma	Anoxia	Anoxia	Anoxia	CV/stroke	CV/stroke
Source	UPenn	UPenn	UPenn	Prodo	Prodo	COH
Use in study	Acetyl-Lys	Acetyl-Lys	Acetyl-Lys	Acetyl-Lys	Acetyl-Lys	Acetyl-Lys

ND, not determined; CV, cardiovascular; Prodo, Prodo Labs Human Islet Isolation Center; UPenn, University of Pennsylvania; COH, Southern California Islet Cell Resource Center at City of Hope.

Supplemental Table 4. RT-qPCR primer sequences.

mRNA	F Primer	R Primer
Sirt2	GCACCTTCTACACATCACACT	ACACGATATCAGGCTTTACCAC
Glp1r	ACGGTGTCCCTCTCAGAGAC	ATCAAAGGTCCGGTTGCAGAA
Tbp	GAAGCTGCGGTACAATTCCAG	CCCCTTGTACCCTTCACCAAT
