# Supplementary figures and images for: The protein deacetylase SIRT2 exerts metabolic control over adaptive β cell proliferation
Source: J Clin Invest. 2025 Jul 31;135(19):e187020. doi: 10.1172/JCI187020 (PMC12483614; doi:10.1172/JCI187020)

IB: Sirt2

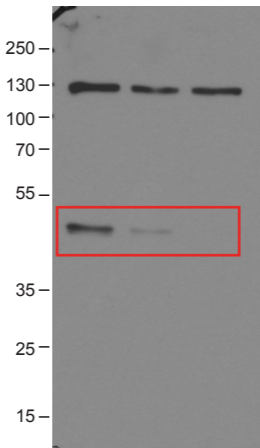

IB:  $\beta$ -tubulin

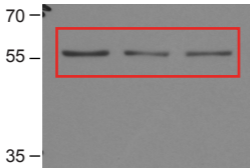

Supplement: Unedited blot and gel images [file jci-135-187020-s238.pdf]
